# Structural basis for itraconazole-mediated NPC1 inhibition

**DOI:** 10.1038/s41467-019-13917-5

**Published:** 2020-01-09

**Authors:** Tao Long, Xiaofeng Qi, Abdirahman Hassan, Qiren Liang, Jef K. De Brabander, Xiaochun Li

**Affiliations:** 1 Department of Molecular Genetics, University of Texas Southwestern Medical Center, Dallas, TX 75390 USA; 2 Department of Biochemistry, University of Texas Southwestern Medical Center, Dallas, TX 75390 USA; 3 Department of Biophysics, University of Texas Southwestern Medical Center, Dallas, TX 75390 USA

**Keywords:** Biophysics, Cell biology, Structural biology

## Abstract

Niemann-Pick C1 (NPC1), a lysosomal protein of 13 transmembrane helices (TMs) and three lumenal domains, exports low-density-lipoprotein (LDL)-derived cholesterol from lysosomes. TMs 3–7 of NPC1 comprise the Sterol-Sensing Domain (SSD). Previous studies suggest that mutation of the NPC1-SSD or the addition of the anti-fungal drug itraconazole abolishes NPC1 activity in cells. However, the itraconazole binding site and the mechanism of NPC1-mediated cholesterol transport remain unknown. Here, we report a cryo-EM structure of human NPC1 bound to itraconazole, which reveals how this binding site in the center of NPC1 blocks a putative lumenal tunnel linked to the SSD. Functional assays confirm that blocking this tunnel abolishes NPC1-mediated cholesterol egress. Intriguingly, the palmitate anchor of Hedgehog occupies a similar site in the homologous tunnel of Patched, suggesting a conserved mechanism for sterol transport in this family of proteins and establishing a central function of their SSDs.

## Abstract (web summary)

Niemann-Pick C1 (NPC1) exports low-density-lipoprotein (LDL)-derived cholesterol from lysosomes and comporses a Sterol-Sensing Domain (SSD). Here authors report a cryo-EM structure of human NPC1 bound to itraconazole which reveals how this binding site in the center of NPC1 blocks a putative lumenal tunnel linked to the SSD.

## Introduction

Cholesterol is indispensable for maintaining the rigidity of cell membranes and producing steroid hormones and bile acids in animal cells. The low solubility of cholesterol in aqueous environments necessitates its transport by dedicated proteins and its insertion in membrane bilayers. Cells employ a number of membrane-embedded proteins containing a conserved five-transmembrane helix (TM) bundle called a Sterol-Sensing Domain (SSD) that facilitates sterol trafficking, regulates sterol biosynthesis, transduces sterol signals, and monitors sterol concentrations^1–4^. These proteins include Niemann-Pick C1 (NPC1); HMG-CoA reductase (HMGR), the rate-controlling enzyme in cholesterol biosynthesis; PATCHED1 (PTCH1), a signal transducer for the Hedgehog pathway; and SCAP, an escort protein for sterol regulatory element binding proteins (SREBPs) (Supplementary Fig. 1). Although near-atomic structures of NPC1 and PTCH1 have been determined^5–9^, the specific mechanism of SSD-mediated cholesterol regulation is not known.

**Fig. 1:**
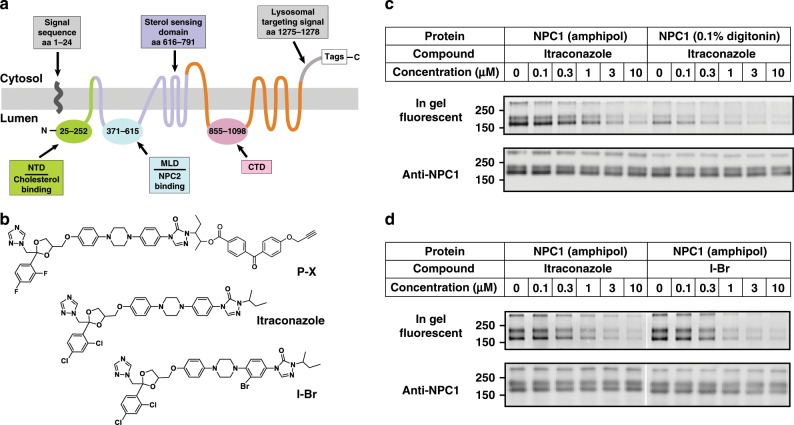
Identification of the interaction between NPC1 and itraconazole. **a** The topology of human NPC1. Functional domains are shown in different colors. NTD, N-terminal domain; MLD, middle lumenal domain; CTD, C-terminal domain. **b** Chemical structures of compounds used in this study. **c**, **d** P-X cross-linking to purified NPC1. **c** Detergent interferes with cross-linking of P-X to purified NPC1. **d** Br-labeled itraconazole (I-Br) competes with cross-linking of P-X to purified NPC1 as well as itraconazole. In **c**, **d**, the proteins were subjected to fluorescent labeling using click chemistry, followed by 8% SDS/PAGE and in-gel fluorescence scanning (Upper) or immunoblotting with anti-NPC1 (Lower). Source data are provided as a Source Data file.

Animal cells acquire cholesterol through receptor-mediated uptake of plasma LDL^10^. Two lysosomal proteins, NPC1 and NPC2, collaborate to export cholesterol from the lysosomal membrane. Human NPC1 has 1278 amino-acid residues, including an N-terminal lumenal domain (NTD), a middle lumenal domain (MLD), a C-terminal lumenal domain (CTD), and 13 TMs (Fig. 1a). When LDL particles are disassembled in the lysosomal lumen, NPC2, a soluble Ig fold-like protein, captures the sterol moiety of cholesteryl ester^11^, allowing the digestion of the lumen-exposed fatty acid chain by acid lipase^10^. Then, NPC1 employs its NTD and MLD to bind NPC2 and transfer its cholesterol^12–15^. This transfer allows cholesterol to be transported across the glycocalyx and to enter the lysosomal membrane^16,17^. Dysfunctional NPC1 and NPC2 fail to export cholesterol, causing accumulation of cholesterol and other lipids in the lysosomal lumen, which leads to Niemann-Pick Type C disease, a fatal lysosomal lipid storage disease^18^.

Previous biochemical and cell biological studies showed that a number of small molecules including cholesterol derivatives, the cationic sterol U18666A, and a class of triazole antifungal drugs bind to NPC1^19–22^. Notably, this binding is retained even after deletion of the NTD^21,22^, which contains a cholesterol-binding site^23,24^, suggesting that there is another ligand-binding site able to accommodate these molecules. Previous studies also showed that U18666A and triazoles block NPC1-mediated cholesterol export and that a point mutation (P691S) in the NPC1-SSD can prevent binding of these ligands^19,22^, implying that this binding site may reside in the SSD. However, it remains unknown how these inhibitors bind to NPC1 and abolish its activity for cholesterol transport. More importantly, this mechanism may answer the fundamental question of whether the SSD performs similar functions in sterol biosynthesis, trafficking, and signaling.

In this paper, we determined the structure of itraconazole-bound human NPC1 at 4-Å resolution revealing that itraconazole binds to the center of NPC1 protein. The functional analysis validates this binding site and shows that itraconazole can block the putative sterol tunnel of NPC1 to inhibit its activity. This finding is consistent with our previous observation on palmitate-mediated PTCH1 inhibition.

## Results

### NPC1 binds to itraconazole in detergent-free solution

Previous studies showed that a photoactivatable derivative of posaconazole (P-X) (Fig. 1b) can be cross-linked to NPC1 in cultured cells when added at nanomolar concentration^22^. This cross-linking is inhibited by itraconazole (Fig. 1b) and U18666A, both of which inhibit cholesterol export from lysosomes^19,22^. P-X also cross-linked to purified NPC1 that was reconstituted in lipid nanodiscs^22^, but not when the protein was solubilized in digitonin (Fig. 1c).

To determine the binding site for itraconazole on NPC1, we expressed and purified the NPC1 protein using n-dodecyl β-D-maltoside (DDM) as the detergent^25^. The protein was then incorporated into amphipol A8-35 and the detergent was removed with bio-beads and further separated by gel filtration in phosphate buffered saline (PBS). Cross-linking assays showed that P-X can cross-link to amphipol-reconstituted NPC1 in PBS without detergent and this cross-linking was competed by added itraconazole (Fig. 1c).

### Itraconazole *cis*-stereoisomers for structural determination

Itraconazole has three chiral centers (Supplementary Fig. 2a). The pharmaceutical form contains four *cis*-stereoisomers in a 1:1:1:1 molar ratio (Supplementary Fig. 2a–d). To determine the activity of the *cis*-stereoisomers, the individual isomers were isolated and identified based on their optical rotation in chloroform following a previous report (Supplementary Fig. 2e)^26^. Then, they were separately tested in a cholesterol re-esterification assay used to measure NPC1-mediated export of cholesterol from lysosomes^27^. Briefly, CHO-7 cells in the medium are deprived of cholesterol to induce the LDL receptors biosynthesis. The cells are then incubated with fetal calf serum (FCS) that contains LDL. The LDL-derived cholesterol will now be transported by functional NPC1 protein from the lysosome to the endoplasmic reticulum (ER). The acyl-CoA:cholesterol actyltransferase named ACAT can now re-esterify the cholesterol in the ER. Therefore, the cells were pulse-labeled with [^14^C] oleate and we can measure the synthesis of cholesteroyl [^14^C] oleate to evaluate the activity of NPC1 protein. Using this assay, we tested the ability of various itraconazole to inhibit NPC1 activity.

**Fig. 2:**
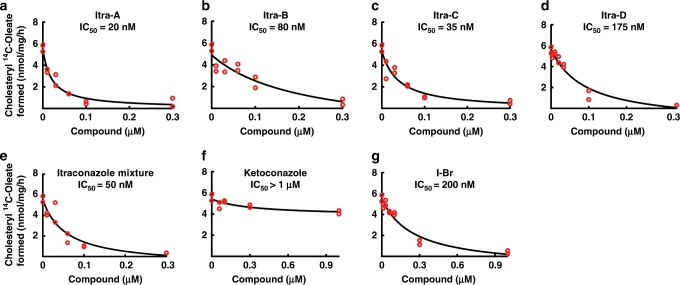
ACAT activity of itraconazole and ketoconazole in CHO-7 cells. On day 0, CHO-7 cells were set up in medium A with 5% LPDS at 2.5 × 10^5^ cells/well in 6-well dish. On day 2, cells were switched to medium A with 5% LPDS containing 50 µM sodium compactin and 50 µM sodium mevalonate. On day 3 the cells received fresh medium B containing compactin and mevalonate with various concentration compounds in the presence of 10% FCS containing lipoproteins. After incubation for 4 h at 37 °C, each cell monolayer was pulse-labeled for 2 h with 0.1 mM sodium [^14^C] oleate (5792 dpm/nmol). The cells were then harvested for measurement of their content of cholesteryl [^14^C] oleate and [^14^C] triglycerides, as described in Methods. The inhibitory constant (IC_50_) values (denoting the concentration at which each compound inhibited cholesterol esterification by 50%) are indicated. Values for incorporation of [^14^C] oleate into [^14^C] triglycerides in cells treated with 0.3 μM of all compounds ranged from 9.7 to 14.3 nmol/h per 1 mg protein. Itraconazole mixture, isomer A (Supplementary Fig. 2a), isomer B (Supplementary Fig. 2b) and isomer C (Supplementary Fig. 2c) were added at final concentrations of 0.3 μM. The isomer D (Supplementary Fig. 2d), I-Br and ketoconazole were added at final concentrations of 1 μM. The biological repeats are shown as individual values. Source data are provided as a Source Data file.

The result shows that the four *cis*-stereoisomers inhibit NPC1-mediated cholesterol transport with IC_50_ values in the range of 20–175 nM. The pharmaceutical mixture of itraconazole showed a IC_50_ value of ~50 nM (Fig. 2a–e, Supplementary Fig. 3)_._ In contrast, ketoconazole did not block the cross-linking of P-X to NPC1^22^, and did not inhibit NPC1-mediated cholesterol transport (Fig. 2f). Importantly, these results demonstrate that all *cis*-stereoisomers are highly active and that a specific stereoisomer is therefore not essential for inhibiting NPC1 activity.

We incubated four *cis*-stereoisomers with purified NPC1 protein individually and we screened these complexes by cryo-EM. The preliminary screening results showed that the NPC1 complex with the *cis*-2*R*,4 *S*,2′*S* isomer (Supplementary Fig. 2d) had the best image contrast and quality for data collection. After 9988 images were collected on a 300 keV Krios electron microscope, the structure was determined at 4.3-Å resolution (Supplementary Fig. 4). An extra density was observed in a hydrophobic cavity which is generated by the SSD, the MLD and the CTD in the center of NPC1 (Supplementary Fig. 4c). However, due to the limited resolution, we could not assign itraconazole unambiguously into the density.

### Structure of itraconazole-bound NPC1

**Fig. 3:**
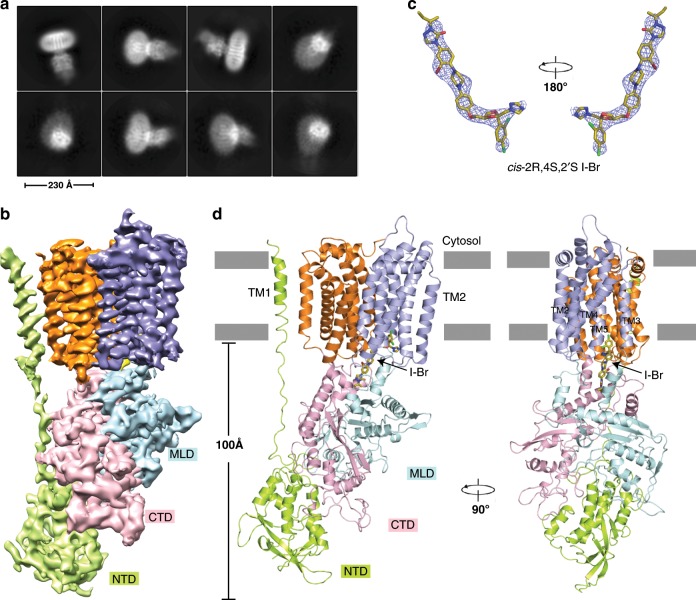
Overall structure of human NPC1 bound to I-Br. **a** Cryo-EM 2D classification from RELION-3. **b** Cryo-EM map after final RELION-3 refinement sharpened using postprocessing. **c** Cryo-EM map of I-Br at 6σ level at 4.0 Å resolution. I-Br is shown as sticks with carbon atoms colored in yellow. **d** Ribbon representation of the structure viewed from the side of the membrane. In this and succeeding figures, NTD and TM1, MLD, CTD, TMs 2-7, and TMs 8-13 are shown in green, light cyan, pink, light blue, and orange, respectively.

To enhance the signal of itraconazole in the density map, we synthesized Br-labeled itraconazole (I-Br) which contains four *cis*-stereoisomers (Supplementary Fig. 5). Our cross-linking assays showed that I-Br binds to NPC1 in a similar manner to the itraconazole mixture (Fig. 1d), and the cholesterol re-esterification assay shows that I-Br inhibits NPC1-mediated cholesterol egress from lysosomes at a IC_50_ value of ~200 nM (Fig. 2g and Supplementary Fig. 3). We collected 7546 images of NPC1 complex with I-Br on a 300 keV Krios electron microscope. After 2D classification, the lumenal domains and transmembrane regions became obvious (Fig. 3a). The structure was then determined at 4.0-Å resolution (Fig. 3b, Supplementary Figs. 6–8 and Supplementary Table 1) from ~210 k particles. The cryo-EM map shows the distinctive electron density of I-Br allowing us to orient the molecule (Fig. 3c). The isomers are indistinguishable at this resolution and they have similar interactions with the binding pocket; therefore, we built a model using the Br-labeled *cis*-2*R*,4 *S*,2′*S* isomer as a representative of the Br-labeled itraconazole mixture. In the following sections, we refer to this isomer for discussion. Due to the limited resolution of the NTD and some linkers, some residues in these regions have been built as poly-alanine (see the details in Method).

**Fig. 4:**
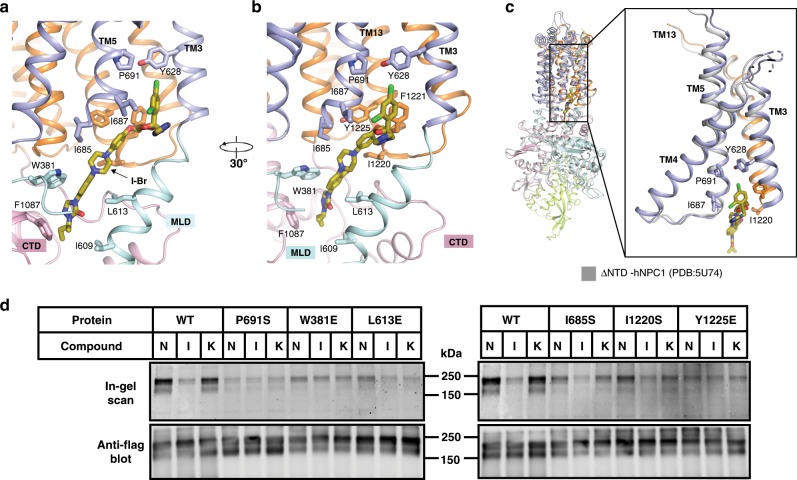
I-Br binds to the hydrophobic cavity of NPC1. **a**, **b** Details of I-Br binding to NPC1. The residues involved in the interaction are shown in sticks; the secondary structures are labeled. **c** Structural comparison of I-Br-bound NPC1 to ΔNTD-NPC1 (gray). **d** Cross-linking of P-X to NPC1 and mutants in intact cells. Compound: none (N), itraconazole (I) and ketoconazole (K). P-X can easily cross-link to WT NPC1, and itraconazole inhibits this reaction but ketoconazole does not. All the mutants have a decreased ability to cross-link to P-X. Source data are provided as a Source Data file.

The I-Br binds to a hydrophobic cavity that is generated by the SSD, the MLD and the CTD in the center of NPC1 (Fig. 3d). Residues in the N-terminal loop and C-terminal helices of the MLD and the CTD, as well as TMs 3, 4, 5, and 13 contribute to the cavity that accommodates I-Br (Figs. 3d, 4a, b). The terminal sec-butyl moiety of I-Br inserts into the cavity and is contacted by residues W381, I609, L613, I685, F1087, I1220, and Y1225 (Fig. 4a, b). Superimposing the cryo-EM structure of I-Br-bound NPC1 to the crystal structure of NPC1-ΔNTD reveals subtle differences between the TMs and no noticeable conformation changes between lumenal domains (Fig. 4c). To validate our structural observation, we have generated five NPC1 mutants (W381E, L613E, Y628S, I685S, and Y1225E) located in the binding site and individually transfected the mutant cDNAs into HEK293S cells. Unlike wild-type NPC1, P-X was not efficiently cross-linked to these mutants in cells (Fig. 4d), supporting our observed itraconazole-binding site.

### Structural comparison with PTCH1-Hedgehog complex

**Fig. 5:**
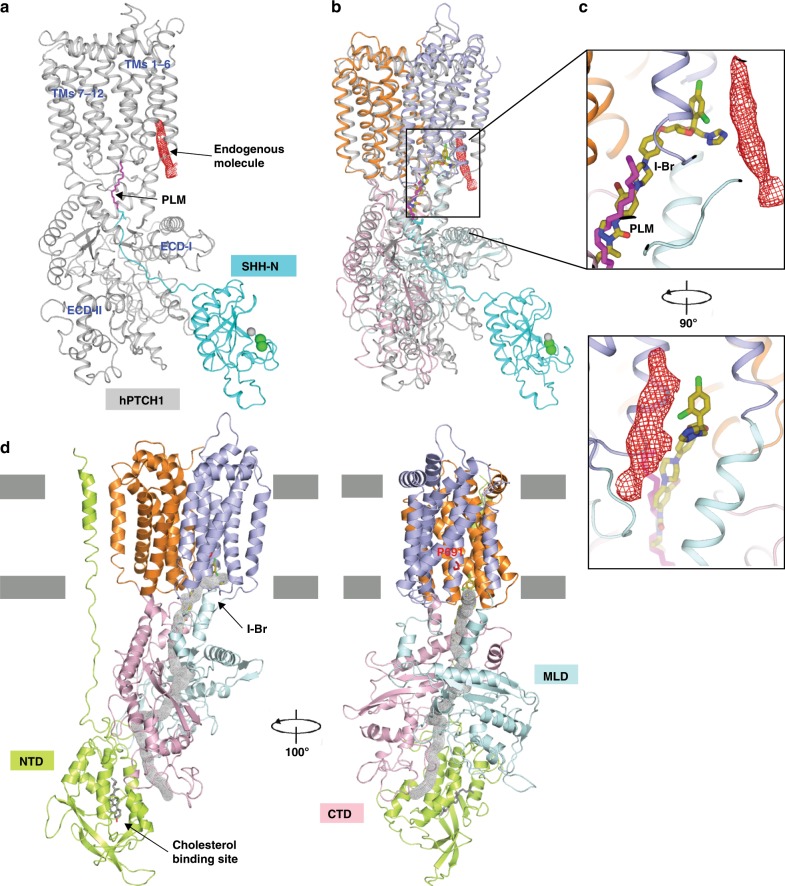
The itraconazole-binding site on NPC1 resembles the palmitate binding site in PTCH1. **a** The overall structure of the palmitoylated, Hedgehog-bound PTCH1 structure (PDB: 6E1H). Palmitate (PLM) is shown as magenta sticks and the endogenous molecule density in the SSD is shown as red mesh, calcium and zinc as green and gray spheres, respectively. **b** Structural comparison of NPC1 and PTCH1. **c** The itraconazole and palmitate bind to a similar cavity of NPC1 and PTCH1. **d** Structure of the I-Br bound NPC1 with the putative tunnel in gray; the I-Br and residue Pro691 are indicated.

PTCH1, a structural homolog of NPC1, has two extracellular domains (ECD-I and ECD-II) and 12 TMs (Fig. 5a). Structural comparison reveals that the transmembrane domain of PTCH1 can be superimposed with TMs 2–13 of NPC1 with an RMSD of 3 Å for 810 Cα atoms, while ECD-I and ECD-II share a similar overall fold with the MLD and CTD of NPC1, respectively (Fig. 5b). Our previous structural studies showed that an endogenous molecule, likely a sterol derivative, binds the SSD of PTCH1 (Fig. 5a). This is consistent with functional data suggesting that PTCH1 may act as a transporter for a sterol^9^ and its SSD could be a part of a transport pathway^8^. Itraconazole binds to the cavity in the center of NPC1 and extends to the pocket of the SSD where a sterol-like density was observed in PTCH1 (Fig. 5c).

The binding site for itraconazole in the center of NPC1 resembles the site at which the palmitate of Hedgehog binds to PTCH1 (Fig. 5b). Previous cell biological and animal studies demonstrated that palmitate modification is essential to release the repressive effect of PTCH1 on Smoothened to activate Hedgehog signaling^7,28,29^, while a deficient palmitate modification causes abnormal development^30,31^. Our previous analysis revealed an internal tunnel with a length of over 100 Å and a minimum diameter of 8 Å, which extends through PTCH1. This tunnel can be blocked by palmitate, which would inhibit the putative transport activity of PTCH1 and cause the accumulation of sterol substrates, most likely cholesterol or its derivatives, to stimulate Hedgehog signaling^8,9,32,33^.

### A tunnel in NPC1 may be a route for cholesterol transport

Calculations using the program MOLE confirm a ~120-Å long tunnel in NPC1 with a minimum diameter of 7 Å, extending from the bottom of the MLD and CTD to the SSD (Fig. 5d). The SSD may be the exit of this tunnel to allow cholesterol to transfer to the lysosomal membrane. Computational studies predicted several putative sterol-binding sites in this tunnel^34^. These findings suggest that NPC1 may employ this tunnel to export cholesterol.

We previously provided mutational evidence for a tunnel in PTCH-1. We introduced two mutations L427R and F1017E into full-length human PTCH1 to block this tunnel by a salt bridge^8^. Cell biological assays showed that this variant lost the activity to inhibit Hedgehog signaling. This evidence supported our hypothesis that the palmitate blocks the activity of PTCH1 by blocking its tunnel^8^. Our current observation, along with previous structural analyses on PTCH1, suggests that NPC1 may employ a similar tunnel to transport LDL-derived cholesterol from the lumen to the lysosomal membrane. Itraconazole may prevent this transport through blocking the tunnel directly.

**Fig. 6:**
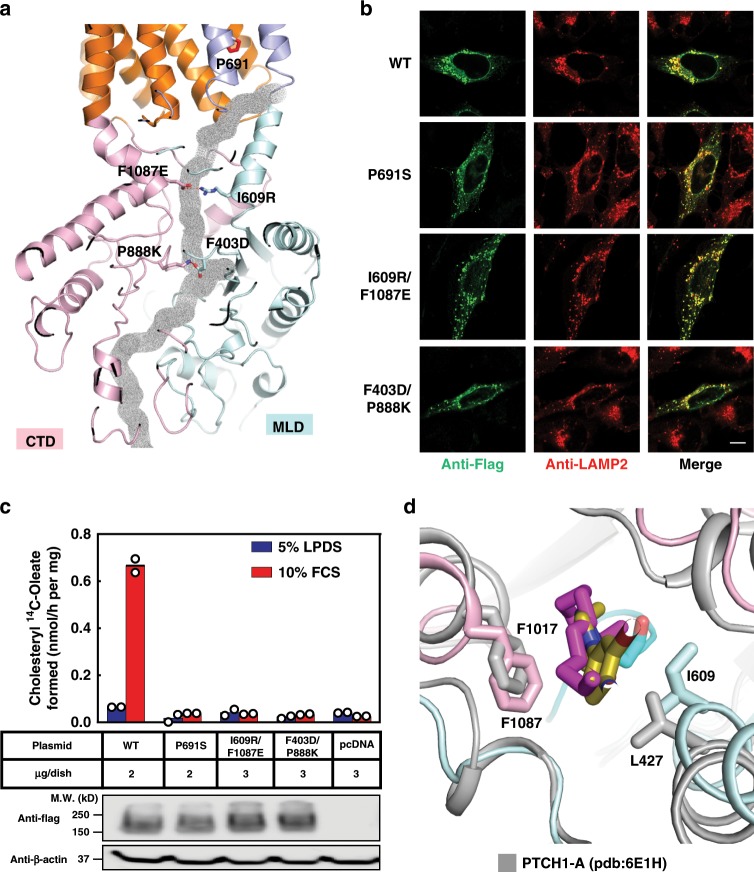
Blocking the putative tunnel of NPC1 abolishes NPC1-mediated LDL-derived cholesterol transport. **a** The distribution of mutations used for blocking the putative tunnel. **b** Localization of NPC1 proteins to lysosomes. SV589 cells were transfected with the plasmids encoding Flag-tagged NPC1 and mutants as described in Methods. Cells were fixed and double stained with 0.8 µg/ml of rabbit monoclonal anti-Flag IgG (green) together with 1 µg/ml of mouse monoclonal anti-LAMP-2 IgG (red), and images were merged (yellow). LAMP-2 is a marker for lysosomes. Immunofluorescence microscopy was performed as described in Methods. Scale bar, 10 µm. **c** ACAT assays. On day 0, NPC1^−/−^ cells were set up in medium A with 5% FCS at 2.5 × 10^5^ cells/60 mm dish. On day 2, monolayers were switched to fresh medium A with 5% LPDS and then transfected with the indicated plasmids encoding NPC1 or mutants using FuGENE HD as the transfection agent. After incubation for 24 h, cells were switched to medium A with 5% LPDS containing 50 µM sodium compactin and 50 µM sodium mevalonate. On day 4, the cells received fresh medium B containing compactin and mevalonate in the presence of either 5% LPDS or 10% FCS containing lipoproteins. After incubation for 4 h at 37 °C, each cell monolayer was pulse-labeled for 2 h with 0.1 mM sodium [^14^C] oleate (6210 dpm/nmol). The cells were then harvested for measurement of their content of cholesteryl [^14^C] oleate and [^14^C] triglycerides as described in Methods. Each bar indicates the mean of duplicate incubations with individual values shown. The mean cellular content of [^14^C] triglycerides in the presence of FCS was not significantly different in cells transfected with pcDNA3.1-NPC1, or its mutants including P691S, I609R/F1087E, F403D/P888K (10.5, 10.5, 11, 10 nmol per hr/mg protein, respectively). The bottom panel shows immunoblots of whole cell extracts (40 μg/lane) using 1:1000 dilution of anti-Flag and 1:5000 dilution of anti-β-actin. **d** Structural comparison of itraconazole-bound NPC1 (colored) and PTCH1-A (gray). The conserved residues are indicated. Palmitate of Hedgehog is shown as magenta sticks. Source data are provided as a Source Data file.

Evidence for a similar tunnel in NPC1 is supported by a study of a P691S mutation found in a patient with NPC disease. NPC1-P691S correctly localizes to the lysosomal membrane but it does not export cholesterol^35^. Our structural analysis reveals that P691 is located at the exit of the putative transport tunnel (Figs. 5d and 6a). The P691S mutation may disrupt the hydrophobic environment of the exit, presumably obstructing the exit of cholesterol from the tunnel to the lysosomal membrane.

To validate the function of this putative tunnel, we generated two NPC1 mutants (I609R/F1087E and F403D/P888K), each of which is predicted to create an artificial salt bridge that should block the tunnel (Fig. 6a). We transfected the variants into *NPC1*^−/−^ cells and employed the cholesterol re-esterification assay to test the export of cholesterol from lysosomes. Although fluorescent staining of these two mutants and the P691S mutant showed correct localization to the lysosome (Fig. 6b), the cholesterol re-esterification assays show that I609R/F1087E and F403D/P888K mutants present minimal activity (Fig. 6c, Supplementary Fig. 9). Notably, I609/F1087 of NPC1 are in a similar position as L427/F1017 of PTCH1 (Fig. 6d). We believe that these amino acids help to create a hydrophobic tunnel that is traversed by sterols.

## Discussion

In this study, we report the cryo-EM structure of itraconazole-bound NPC1. Itraconazole binds to a cavity, which is created by the MLD and the CTD in the center of NPC1, with the head portion containing the dichlorophenyl moiety of itraconazole extending into the transmembrane region of NPC1, where it forms several hydrophobic contacts with the NPC1-SSD. Ketoconazole, which has a shorter tail portion than that of itraconazole^22^, could not inhibit the cross-linking between P-X and NPC1 (Fig. 4d) presumably due to a lack of enough contacts in the cavity of NPC1. Notably, this binding mode resembles the interaction of Hedgehog palmitate with its receptor PTCH1. Previous studies have shown that the palmitate modification of Hedgehog is necessary to repress the activity of PTCH1 and consequently trigger Hedgehog signaling^7,28–31^. Similarly, itraconazole may bind NPC1 and prevent NPC1-mediated LDL-derived cholesterol export, causing its accumulation in the lysosomal lumen. These discoveries imply a putative mechanism of SSD-mediated sterol transport.

In the cryo-EM structures of PTCH1, a sterol-like density appears in the SSD^5,7–9^, the location of which corresponds to the binding site of the dichlorophenyl moiety of itraconazole in NPC1 (Fig. 5c). Because NPC1 and PTCH1 are structural homologues, NPC1 may accommodate a sterol-like molecule as well. In both NPC1 and PTCH1, there is *a* > 100 Å-long tunnel through the protein from the lumenal/extracellular domains to the SSD. Several studies indicate that NPC1 and PTCH1 may function as sterol transporters^9,36^. Therefore, it is tempting to speculate that the SSD may serve as a part or a regulator of the sterol tunnel, and that either itraconazole or palmitate can occupy this tunnel to inhibit the sterol transport activity. Cholesterol occupancy in the SSD pocket might in turn be regulated by the concentration of free cholesterol or its derivatives in the lipid bilayer. It is possible that occupancy of this pocket allosterically affects the conformation of NPC1 or PTCH1 to regulate its transport activity.

**Fig. 7:**
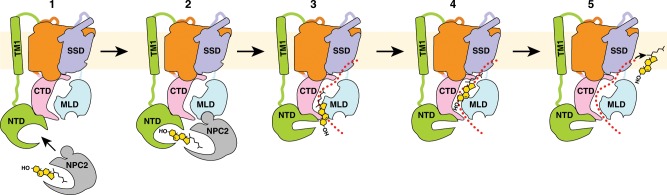
A working model of NPC1/NPC2-mediated LDL-derived cholesterol transport out of lysosomes. NPC2 protein is shown in gray, cholesterol is shown in yellow, and the putative tunnel of NPC1 is indicated by the red dashed line.

Based on previous studies and our current observation, we propose a mechanism of NPC1-mediated cholesterol transport (Fig. 7). NPC2 captures the four-ring moiety and the isooctyl chain of the cholesteryl ester by its hydrophobic pocket. After lipase removes the fatty acid chain on the 3′ hydroxyl group of the cholesteryl ester, the NPC2-cholesterol complex binds to the MLD of NPC1^12,14^. Since the on-off rate of NPC2 to cholesterol is higher than that of NPC1-NTD^13^, cholesterol can be delivered from NPC2 to NPC1-NTD through a two-pocket tunnel^14^. Structural analysis reveals that the cholesterol-binding pocket of the NTD is not aligned with the putative tunnel of NPC1 (Fig. 5d). After this transfer, the NTD must therefore change its conformation, connecting the NTD pocket to the entrance of the NPC1 internal tunnel, allowing cholesterol to be transported throughout the entire NPC1 protein. A previous study has shown that the NTD of one full-length NPC1 molecule can transfer cholesterol to another NPC1 molecule without the NTD^37^. The poly-proline linker provides flexibility between the NTD and TM1 and this may allow the NTD to orient so that it can deliver cholesterol to the entrance of the tunnel of an adjacent NPC1 molecule.

The mechanism proposed in Fig. 7 provides a double transfer mechanism to assure that cholesterol is presented to the lysosomal membrane with its hydrophobic side chain leading and its polar hydroxyl group following. First, as previously demonstrated the hydrophobic isooctyl side chain of cholesterol binds to NPC2, leaving the hydroxyl exposed^11^. When cholesterol is transferred to the NTD, the hydroxyl binds to the NTD, leaving the isooctyl side chain exposed^17^. We speculate that this orientation reverses again when cholesterol transfer from NTD to the tunnel, thereby allowing the isooctyl side chain to lead the way. While this manuscript was under review, a crystal structure of yeast NPC1 revealed an ergosterol in the putative tunnel^38^. The isooctyl side chain of ergosterol faces to the membrane supporting our speculation (Supplementary Fig. 10). More importantly, in this crystal structure, the sterol-binding pocket of NPC1-NTD is aligned to the lumenal tunnel suggesting a direct transfer between the NTD and the tunnel. These findings suggest that the transfer of cholesterol to the NTD allows the orientation to reverse, since NPC2 cannot transfer its cholesterol directly to the tunnel—the sterol would be in the wrong orientation. Remarkably, this tunnel might not provide enough space for cholesterol to turn around. It maintains the orientation that allow its insertion into the lysosomal membrane properly and efficiently.

## Methods

### Cell culture and plasmid constructions

Medium A is a 1:1 mixture of Ham’s F-12 medium and Dulbecco’s modified Eagle’s medium (DMEM) containing 2.5 mM L-glutamine. Medium B is L-glutamine-free DMEM. Medium C is DMEM-low glucose (1000 mg/l). All media contained 100 units/ml penicillin and 100 µg/ml streptomycin sulfate unless otherwise noted. NPC1^−/−^ cells (previously referred to as 10–3 cells) are a stable line of mutant CHO-K1 cells (ATCC, CCL-61) that lack detectable NPC1^36^ which were a gift from Dr. Laura Liscum’s lab at Tufts University. These NPC1^−/−^ cells were grown in medium A with 5% FCS. CHO-7 cells, a clone of CHO-K1 cells that were selected for growth in lipoprotein-deficient serum (LPDS)^39^ were made by Drs. Brown and Goldstein’s lab in our department. These selected cells were grown in medium A with 5% LPDS. SV589 cells (Human Genetic Cell Repository, GM 639) are a line of SV40-immortalized human fibroblasts^40^. These cells were grown in medium C with 5% FCS. Stock cultures of all cell lines were maintained in monolayer culture at 37 °C in an 8.8% CO_2_ incubator except for the SV589 cells, which were maintained at 5% CO_2_. All cell lines were routinely monitored for mycoplasma contamination.

pNPC1 encoding wild-type human NPC1 (amino acids 1–1278) followed sequentially by three tandem copies of the Flag epitope tag (DYKDDDDK) was constructed into pcDNA3.1. Expression is achieved with the cytomegalovirus (CMV) promoter. Point mutations were introduced into the coding region of pNPC1 by site-directed mutagenesis using the QuikChange II XL Site-Directed Mutagenesis Kit (Agilent Technologies, Santa Clara, CA) and primers which are indicated in Supplementary Table 2. The coding region of each plasmid was sequenced to ensure integrity of the construct.

### Cross-linking

Itraconazole and ketoconazole were obtained from Sigma-Aldrich, and *cis*-stereoisomers of itraconazole were obtained from WuXi AppTec Co., Ltd. P-X was synthesized as described previously^22^. All compounds were dissolved in DMSO and stored as 10 mM stock solutions in multiple aliquots at −80 °C.

For Cross-Linking of P-X to purified NPC1 protein, each reaction contained 0.5 μg of indicated protein, 0.3 μM P-X, and various amounts of itraconazole in a final volume of 100 μL of buffer containing 50 mM Hepes pH7.5 and 150 mM NaCl with or without 0.1% Digitonin (Calbiochem). After incubation for 1 h at 37 °C, the mixture was subjected to UV cross-linking for 10 min and then tagged with Alexa Fluor 532 by means of copper-catalyzed azide-alkyne cycloaddition (click chemistry)^22,41^. The reactions were carried out by mixing an aliquot of each mixture (50 μL), in a final volume of 58.5 μL, with 3 μL of 1.7 mM Tris[(1-benzyl-1H-1,2,3-triazol-4-yl) methyl] amine (TBTA), 2 μL of 50 mM CuSO4, 1 μL of 50 mM Tris(2-carboxyethyl) phosphine (TCEP), 1.5 μL 10% SDS and 1 μL of 1.25 mM Alexa Fluor 532 azide. The mixture was incubated at room temperature for 1 h.

To visualize the fluorescent-labeled proteins by in-gel fluorescence, 20 μL. aliquots of each sample were mixed with 4x loading dye and subjected to 8% SDS/PAGE, after which the gels were scanned with a Typhoon image scanner (GE Healthcare; filter setting: excitation, 533 nM; emission, 555 nm; high sensitivity). After scanning the gel, the proteins were transferred to nitrocellulose and blotted with rabbit monoclonal anti-NPC1 as indicated in the figure legends.

For cross-linking of P-X to NPC1 and mutants in intact cells, the mammalian HEK-293S GnTI^-^ cells (ATCC, CRL-3022) overexpressing NPC1 and mutants were set up in 500 mL of freestyle medium with 2% FCS at a density of 2 × 10^6^ cells per ml. The cells were transfected with 0.5 mg of the indicated plasmid using PEI (Polysciences) as the transfection agent. After 48 h, cell culture was aliquoted to 10 cm dishes. Each dish received a direct addition of DMSO (final concentration, 0.2%) containing 0.3 μM P-X and 3 μM of various competitor compounds as described in the figure legends. After incubation for 1 h at 37 °C in the dark and without a change of media, the cells were irradiated for 10 min at room temperature under 306-nm UV light (Atlanta Light Bulb Co.) in a UV Stratalinker 2400 apparatus (Stratagene). Cells were subjected to centrifugation at 4000 g for 5 min. The pellet was washed once with PBS and then solubilized with 1 mL of buffer containing 50 mM Hepes pH 7.5, 150 mM NaCl, 1%(wt/vol) n-Dodecyl-β-D-maltopyranoside (DDM, Anatrace). Each solubilized lysate was incubated at room temperature for 1 h, and clarified by centrifugation at 15000 g for 1 min. The supernatant was then transferred to a fresh tube and incubated with anti-FLAG M2 magnetic beads for 1 h. After which the beads were applied to a magnet, washed three times with 1 mL of wash buffer containing 50 mM Hepes pH 7.5, 150 mM NaCl, 0.02% (wt/vol) DDM, and eluted with 100 μL of wash buffer containing 15 μg of 3x FLAG peptide. The purified protein was then tagged with Alexa Fluor 532 using click chemistry as described above. An aliquot of each sample was subjected to 8% SDS/PAGE, and fluorescent-labeled proteins were visualized by in-gel fluorescence as described above. After scanning the gel, the proteins were transferred to nitrocellulose and blotted with mouse monoclonal anti-Flag.

### Protein expression and purification

Human NPC1 protein was expressed and purified as previously described^25^, except for using Strep-tag instead of His-tag. Briefly, the protein was expressed using baculovirus-mediated transduction of mammalian HEK-293S GnTI^−^ cells (ATCC, CRL-3022). At 48 h post infection, the cells were disrupted by sonication in buffer B, containing 20 mM Hepes, pH7.5, 150 mM NaCl with 1 mM PMSF and 5 μg/mL leupeptin. After low speed centrifugation, the resulting supernatant was incubated in buffer B with 1% (w/v) DDM for 1 h at 4 °C. The lysate was centrifuged again, and the supernatant was loaded onto a Strep-Tactin affinity column (IBA). After washing twice, the protein was eluted in 20 mM Hepes, pH7.5, 150 mM NaCl, 2.5 mM desthiobiotin, 0.02% DDM and concentrated. The concentrated protein was further purified by Superdex 200 increase column (GE Healthcare) in buffer B with 0.02% DDM. The peak fractions were mixed with amphipol A8-35 (Anatrace) at 1:3 (w/w) for 4 h. Detergent was removed with Bio-Beads (Bio-Rad) overnight and further separation on a Superdex 200 increase column in PBS (Sigma-Aldrich). The peak fractions were collected and concentrated to 4–6 mg/ml for grid preparation.

### EM sample preparation and imaging

For preparation of the NPC1-itraconazole complex sample, NPC1 (final concentration 25 μM) in amphipols was mixed with itraconazole *cis*-stereoisomers or I-Br (final concentration 100 μM) for 30 min prior to grid preparation. Sample was added to Quantifoil R1.2/1.3 400 mesh Au holey carbon grids (Quantifoil), blotted using a Vitrobot Mark IV (FEI), and frozen in liquid ethane. The grids were imaged in a 300 keV Titan Krios (FEI) with a Gatan K3 Summit direct electron detector (Gatan). Data were collected at 0.66 Å/pixel with a dose rate of 23 electrons per physical pixel per second. Images were recorded for 1.5 s exposures in 30 subframes to give a total dose of 80 electrons per Å^2^.

### Imaging processing and 3D reconstruction

Dark subtracted images were first normalized by gain reference that resulted in a pixel size of 0.66 Å/pixel. Drift correction was performed using the program MotionCor2^42^. The contrast transfer function (CTF) was estimated using CTFFIND4^43^. To generate NPC1 templates for automatic picking, around 3000 particles were manually picked and classified by 2D classification in RELION-3^44^. After auto-picking, the low-quality images and false-positive particles were removed manually. About 1.06 million particles of NPC1 with itraconazole-D were extracted. Two rounds of reference-free 2D classifications were performed and ~785 k particles were selected for 3D classification. We used the cryo-EM structure of human NPC1 which was determined by us with the data collected from a 200 keV Arctica (FEI) low-pass filtered to 60 Å as the initial model for 3D classification. The best class, containing 344,216 particles, provided a 4.8 Å map after 3D auto-refinement without a mask in RELION-3. CTF refinement and Bayesian polishing of particles were then performed on the 344k particles using RELION-3. The 3D refinement using a soft mask and solvent-flattened Fourier shell correlations (FSCs) yielded a reconstruction at 4.36 Å before post-processing. Applying a soft mask in RELION-3 post-processing yielded a final cryo-EM map of 4.28 Å. Resolution was estimated using the Fourier shell correlation (FSC) 0.143 criterion.

After removing low-quality images and false-positive particles, about 645k particles of NPC1 with I-Br were extracted. Three rounds of reference-free 2D classifications were performed and ~440 k particles were selected for 3D classification. We used the cryo-EM structure of human NPC1 which was determined by us with the data collected from a 200 keV Arctica (FEI) low-pass filtered to 60 Å as the initial model for 3D classification. The best class, containing 209,612 particles, provided a 6.4 Å map after 3D auto-refinement without a mask in RELION-3. Bayesian polishing was then performed on the 209k particles in RELION-3. The 3D refinement using a soft mask and solvent-flattened Fourier shell correlations (FSCs) yielded a reconstruction at 4.53 Å before post-processing. Applying a soft mask in RELION-3 post-processing yielded a final cryo-EM map of 4.02 Å. Resolution was estimated using the Fourier shell correlation (FSC) 0.143 criterion.

### Model construction

To obtain better side-chain densities for model building, we sharpened the map of NPC1 with I-Br using post-processing in RELION-3. The structures of NPC1* (PDB: 5U74) and NPC1-NTD (PDB:3GKH) were docked to the map. The model was first manually adjusted and refined using COOT. The densities of residues 23, 288–333, 642–649, 797–813, and 1256–1278 of NPC1 were not resolved nor built. Residues 30, 35, 37–39, 43–45, 129–143, 209–211, 215, 246, 250, 252, 260, 265, 269–274, 276-277, 279-284, and 287 were built with alanine due to limited local resolution. The Br-labeled *cis*-2*R*,4 *S*,2′*S* itraconazole isomer was built into the ligand density.

### Model refinement and validation

The model was refined in real space using PHENIX^45^ and also in reciprocal space using Refmac with secondary-structure restraints and stereochemical restraints^46,47^. For cross-validations, the final model was refined against one of the half maps generated by 3D auto-refine and the model vs. map FSC curves were generated in the Comprehensive validation module in PHENIX. Local resolutions were estimated using RELION-3. PHENIX and MolProbity^48^ were used to validate the final model. Structure Figures were generated using PyMOL (http://www.pymol.org) and Chimera^49^.

### Co-localization by immunofluorescence microscopy

SV589 cells were set up on glass coverslips at 1.5 × 10^5^ cells per six-well plate in 2 ml medium C with 5% FCS. At 24 h after plating, cells were transfected with 1 µg of the indicated plasmid using FuGENE HD as the transfection agent. At 24 h after transfection, cells were fixed for 15 min in 3.7% formaldehyde in PBS at room temperature and permeabilized for 10 min in methanol at −20 °C. After blocking by incubation with 1 mg/ml bovine serum albumin in PBS, cells were double-labeled with mouse monoclonal anti-LAMP-2 (1: 100, BD biosciences, 555803) and rabbit monoclonal anti-Flag (1: 100, Sigma-Aldrich, F7425) followed by goat anti-rabbit IgG conjugated with AlexaFluor 488 (1: 300, Ivitrogen, A-11008) and goat anti-mouse IgG conjugated with AlexaFluor 594 (1: 300, Invitrogen, A-11005). The coverslips were then mounted in Shandon Immu-mount (Thermo Scientific) and fluorescence images were acquired using a Plan-Apochromat ×63/1.4 oil DIC objective (Zeiss, Oberkochen, Germany), a Zeiss LSM 800 microscope (Zeiss), and ZEN imaging software (Zeiss).

### Immunoblot analysis

Whole cell extracts were subjected to electrophoresis in phosphate-buffered saline (PBS) containing 0.3% SDS and a 1:500 dilution of Benzonase Nuclease. Samples were applied to Bolt 4–12% gradient gels. After electrophoresis, the proteins were transferred to nitrocellulose filters, which were then incubated with the indicated primary antibody. Bound antibodies were visualized by chemiluminescence (SuperSignal West Pico Chemiluminescent Substrate, Thermo Scientific, Waltham, MA) after a 1 h incubation with either horse anti-mouse IgG (1: 5000, Cell Signaling, 7076) or goat anti-rabbit IgG (1:5000, Cell Signaling, 7074) conjugated to horseradish peroxidase. The immunoblot using the HRP-conjugated β-actin antibody was visualized without a secondary antibody. The images were scanned using an Odyssey FC Imager (Dual-Mode Imaging System; 2 min integration time) and analyzed using Image Studio ver. 5.0 (LI-COR Biosciences, Lincoln, NE).

### ACAT activity in CHO-7 cell

On day 0, cells were set up in medium A with 5% LPDS at 2.5 × 10^5^ cells/well in six-well dish. On day 2, cells were switched to medium A with 5% LPDS containing 50 µM sodium compactin and 50 µM sodium mevalonate. On day 3 the cells received fresh medium B containing compactin and mevalonate with indicated concentration triazole compounds in the presence of 10% FCS containing lipoproteins. After incubation for 4 h at 37 °C, each cell monolayer was pulse-labeled for 2 h with 0.1 mM sodium [^14^C] oleate (5792 dpm/nmol). The cells were then harvested for measurement of their content of cholesteryl [^14^C] oleate and [^14^C] triglycerides.

### ACAT activity in NPC1^−/−^ cells

On day 0, cells were set up in medium A with 5% FCS at 2.5 × 10^5^ cells/60 mm dish. On day 2, monolayers were switched to fresh medium A with 5% LPDS and then transfected with the indicated plasmids encoding NPC1 and mutants using FuGENE HD as the transfection agent. After incubation for 24 h, cells were switched to medium A with 5% LPDS containing 50 µM sodium compactin and 50 µM sodium mevalonate. On day 4, the cells received fresh medium B containing compactin and mevalonate in the presence of either 5% LPDS or 10% FCS containing lipoproteins. After incubation for 4 h at 37 °C, each cell monolayer was pulse-labeled for 2 h with 0.1 mM sodium [^14^C] oleate (6210 dpm/nmol). The cells were then harvested for measurement of their content of cholesteryl [^14^C] oleate and [^14^C] triglycerides.

### Cholesterol esterification assay

The rate of incorporation of [^14^C] oleate into cholesteryl [^14^C] oleate and [^14^C] triglycerides by monolayers of NPC1^−/−^ cells and CHO-7 cells was measured as described previously^39^. In brief, cells were switched to medium containing LPDS supplemented with compactin and mevolonate at 16 h before the experiments. On the day of the experiment, cells were incubated for 4 h with fresh medium containing FCS as a source of cholesterol-containing lipoproteins. Cells were then pulse –labeled for 2 h with 0.1 mM sodium [^14^C] oleate. Cells were then washed, and the lipids were extracted in hexane:isopropanol (3:2, vol:vol), separated on a silica gel G thin-layer chromatogram (developed in heptane:ethylether:acetic acid, 90:30:1), and quantified by scintillation counting with the use of an internal standard for recovery^27^. The amounts of cholesteryl [^14^C] oleate and [^14^C] triglycerides formed are expressed as nanomoles formed per hour per milligram cell protein.

### Reporting summary

Further information on research design is available in the Nature Research Reporting Summary linked to this article.

## Supplementary information

Supplementary Information

Reporting Summary

## Data Availability

Data supporting the findings of this paper are available from the corresponding author upon reasonable request. A reporting summary for this Article is available as a Supplementary Information file. The source data underlying Figs. 1c, d, 2, 4d, 6c and Supplementary Figs. 3 and 9 are provided as a Source Data file. The 3D cryo-EM density maps have been deposited in the Electron Microscopy Data Bank under the accession numbers EMD-20834. Atomic coordinates for the atomic model have been deposited in the Protein Data Bank under the accession numbers 6UOX.

## Source data

Source Data
