# Supplementary material for: Structural basis for itraconazole-mediated NPC1 inhibition
Source: Nat Commun. 2020 Jan 9;11:152. doi: 10.1038/s41467-019-13917-5 (PMC6952396; doi:10.1038/s41467-019-13917-5)
Supplement: Supplementary file 1 — Supplementary Information [file 41467_2019_13917_MOESM1_ESM.pdf]

Structural basis for itraconazole-mediated NPC1 inhibition

Tao Long, Xiaofeng Qi, Abdirahman Hassan, Qiren Liang, Jef K. De Brabander, and Xiaochun Li

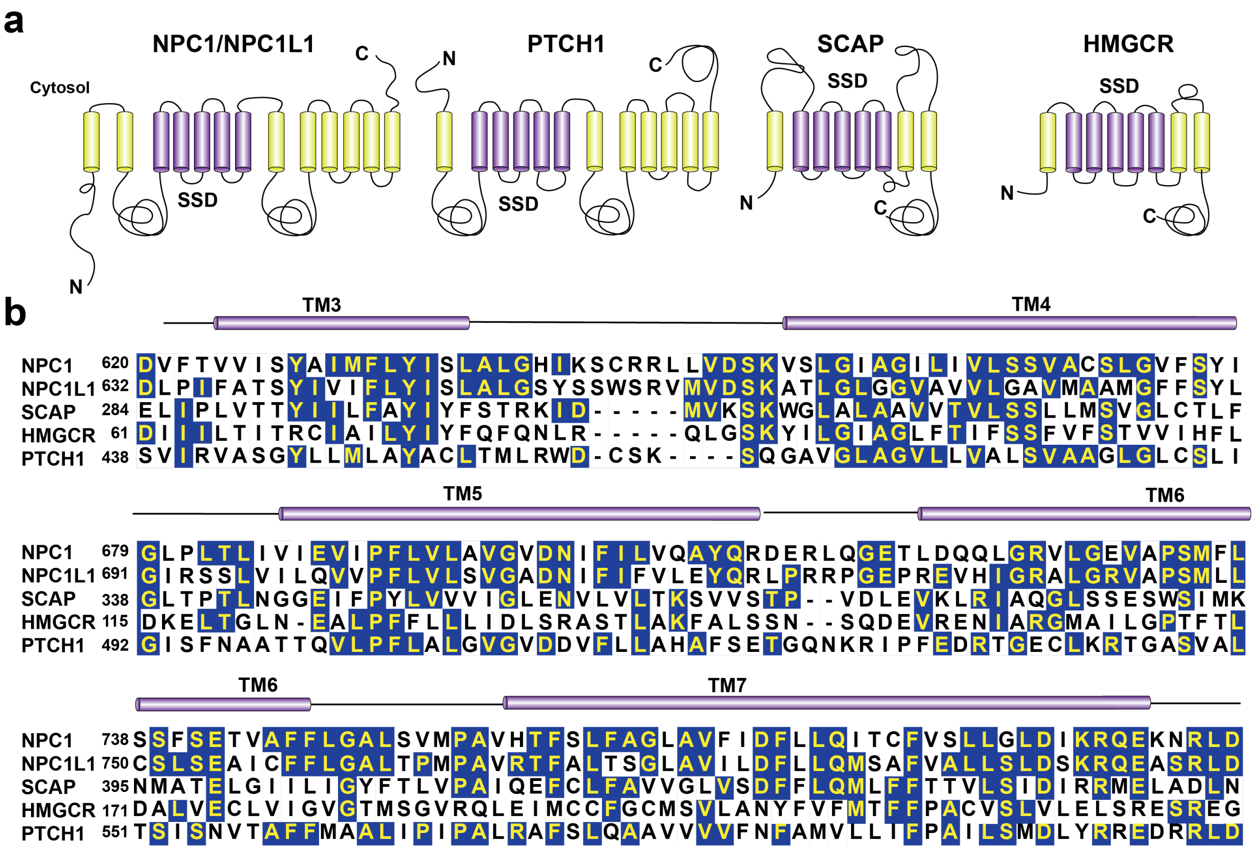

Supplementary Figure 1 Sequence alignment of SSD-containing proteins.

a, The topology of the proteins. The transmembrane regions are shown as columns and the SSD is colored in purple. **b,** Sequence alignment of the SSDs from each protein. The secondary structures of NPC1 are shown above.

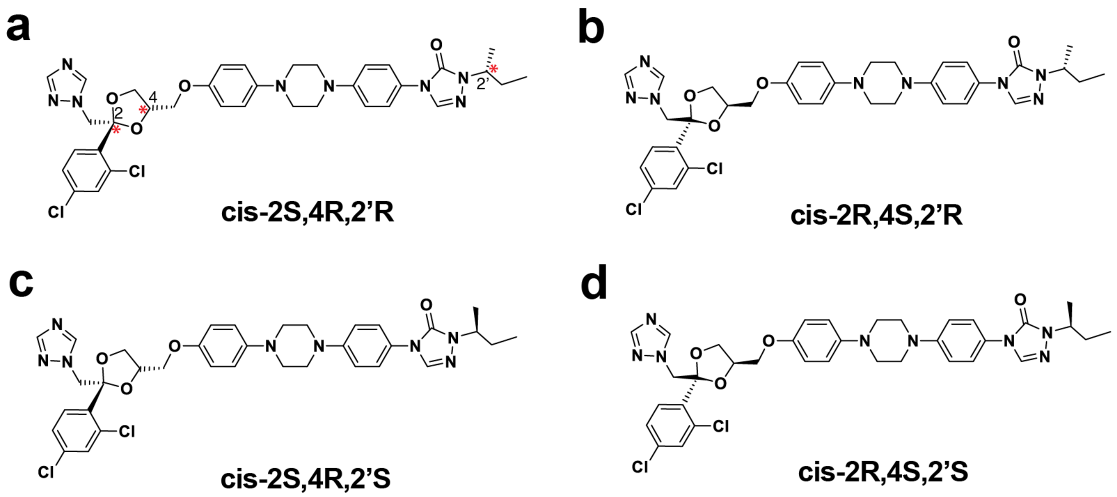

e

Testing Data						Reference Data	
Itroconazole	Wt (mg)	Chloroform (mL)	C (mg/mL)	[α]D		Itroconazole	[α]D
A	4.6	2	2.3	-9.2		cis-2S,4R,2'R	-12.3
B	4.2	2	2.1	+4.3		cis-2R,4S,2'R	+5.7
C	4.2	2	2.1	-3.1		cis-2S,4R,2'S	-5.5
D	4.0	2	2.0	+10.8		cis-2R,4S,2'S	+12.6

Supplementary Figure 2 Identification of *cis*-isomers of itraconazole.

a-d, The structures of four *cis*-isomers of itraconazole. The chiral centers are indicated by the red asterisks. **e,** The identification of itraconazole *cis*-isomers by their optical rotation $[\alpha]_D$ in $CHCl_3$ [1].

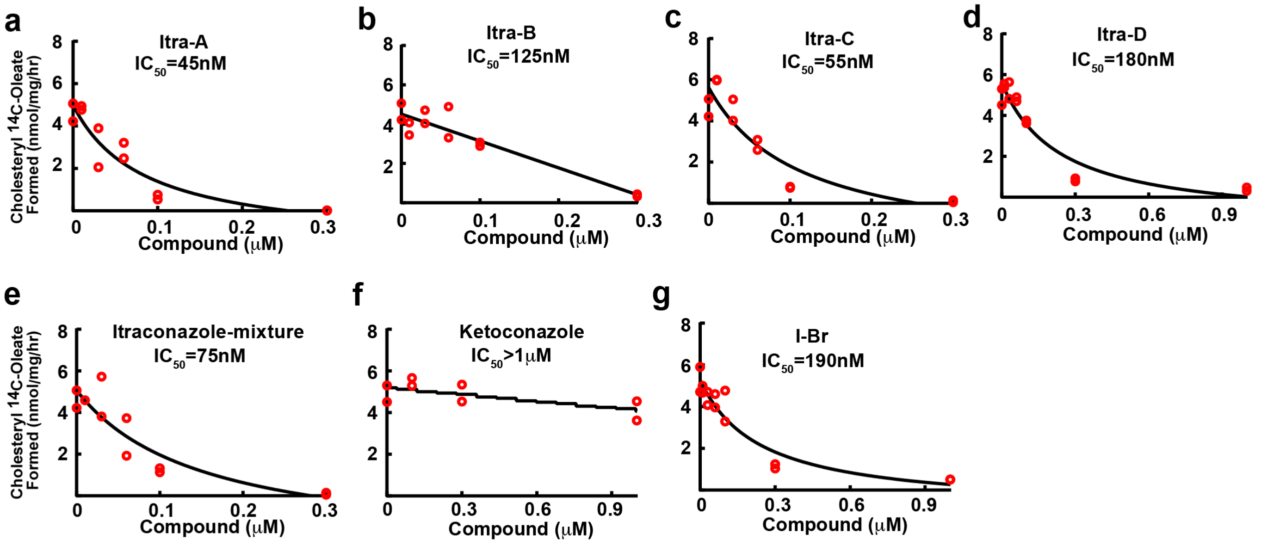

Supplementary Figure 3 Biological repeats of ACAT activity of itraconazole and ketoconazole in CHO-7 cells. Source data are provided as a Source Data file.

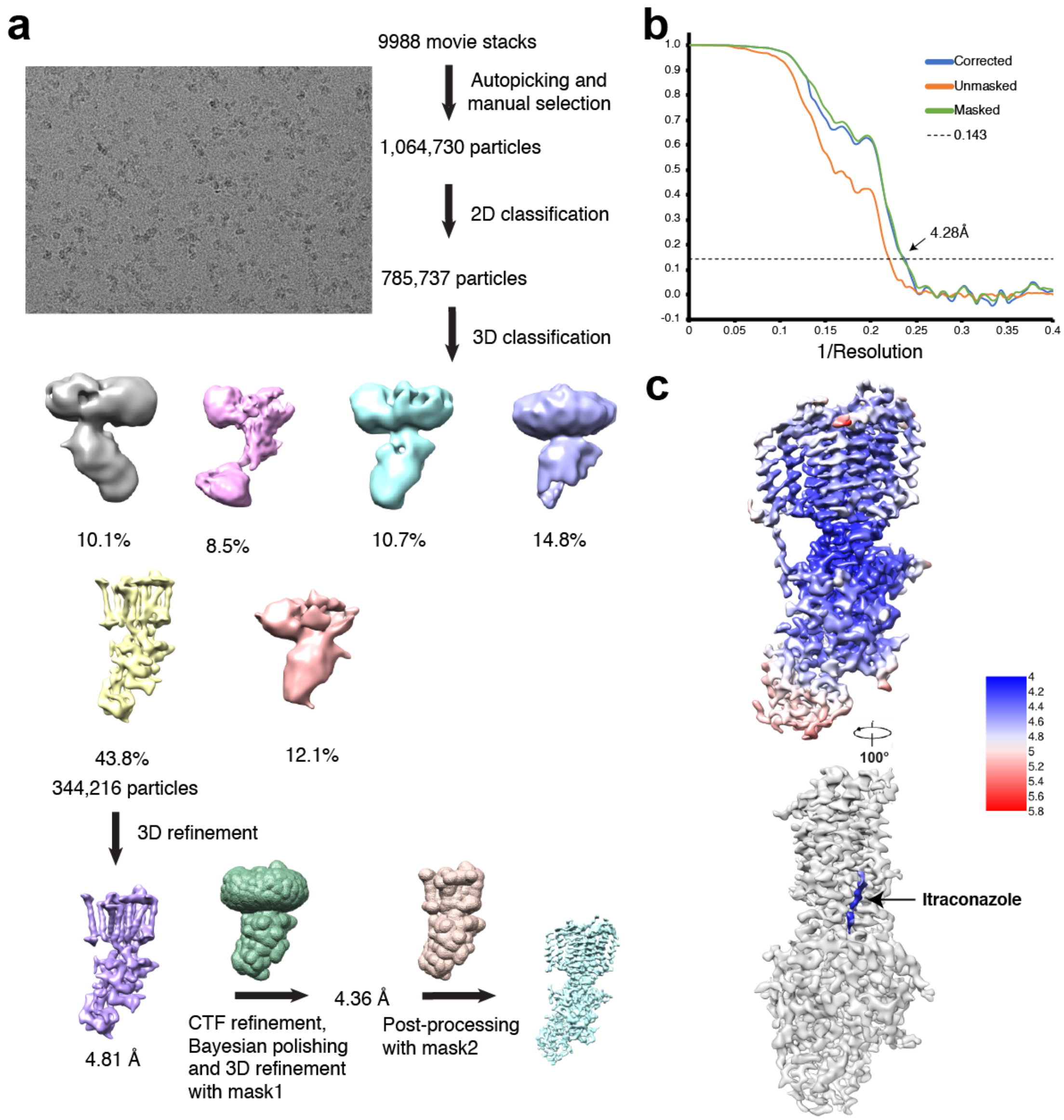

Supplementary Figure 4 Data processing and model quality assessment of NPC1-itraconazole-D.

a, The data processing workflow. A representative electron micrograph at a defocus of −2.0 µm. **b,** FSC curve of the structure as a function of resolution using RELION-3 output. **c,** Density maps of NPC1 structure colored by local resolution estimate using RELION-3.

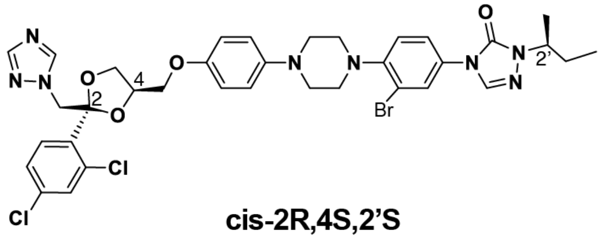

cis-2R,4S,2'S

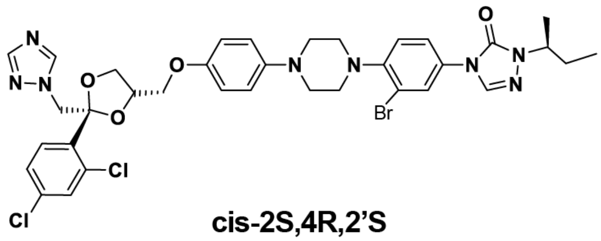

cis-2S,4R,2'S

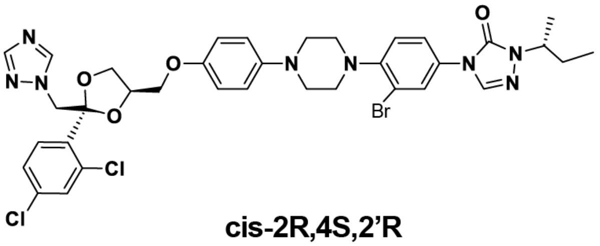

cis-2R,4S,2'R

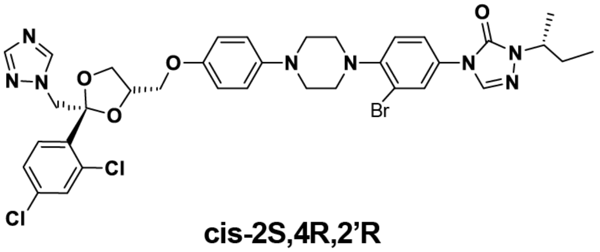

cis-2S,4R,2'R

Supplementary Figure 5 The structures of four isomers of Br-labeled itraconazole.

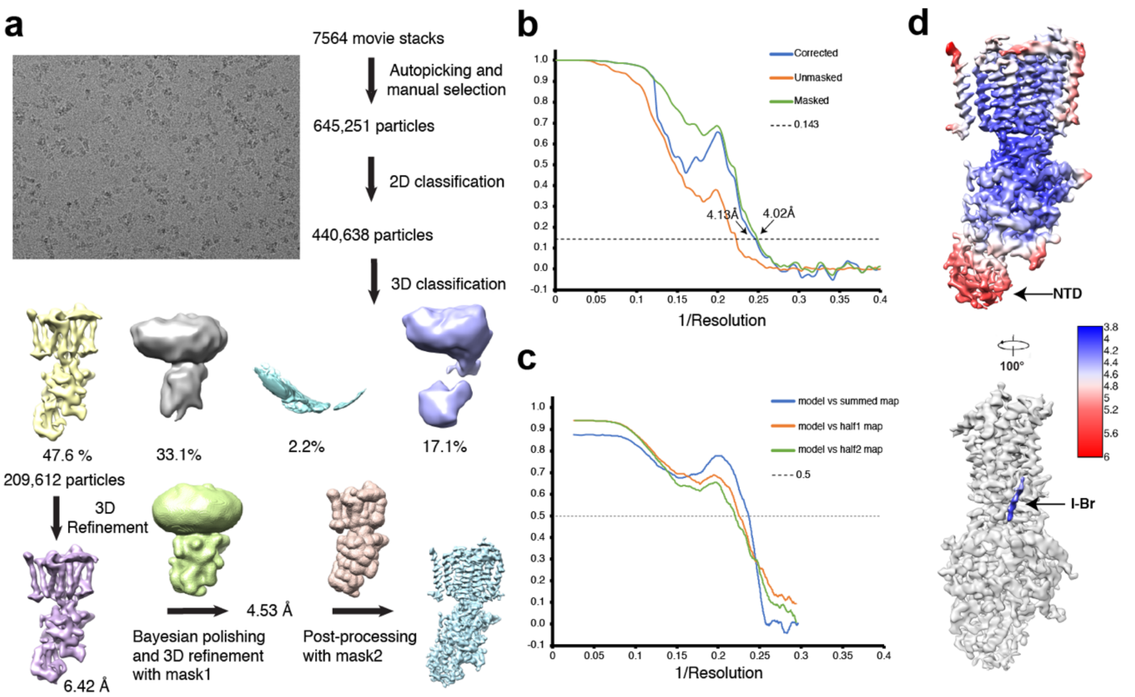

Supplementary Figure 6 Data processing and model quality assessment of I-Br bound NPC1.

a, The data processing workflow. A representative electron micrograph at a defocus of −2.0 µm. **b,** FSC curve of the structure as a function of resolution using RELION-3 output. **c,** FSC curves for cross-validation between the models and the maps. Curves for the final refined model versus the reconstruction from all particles in blue (sum), for the model refined against the reconstruction from only half of the particles versus the same reconstruction in orange (work), and for the same model versus the reconstruction from the other half of the particles in green (free). **d,** Density maps of NPC1 structure colored by local resolution estimate using RELION-3.

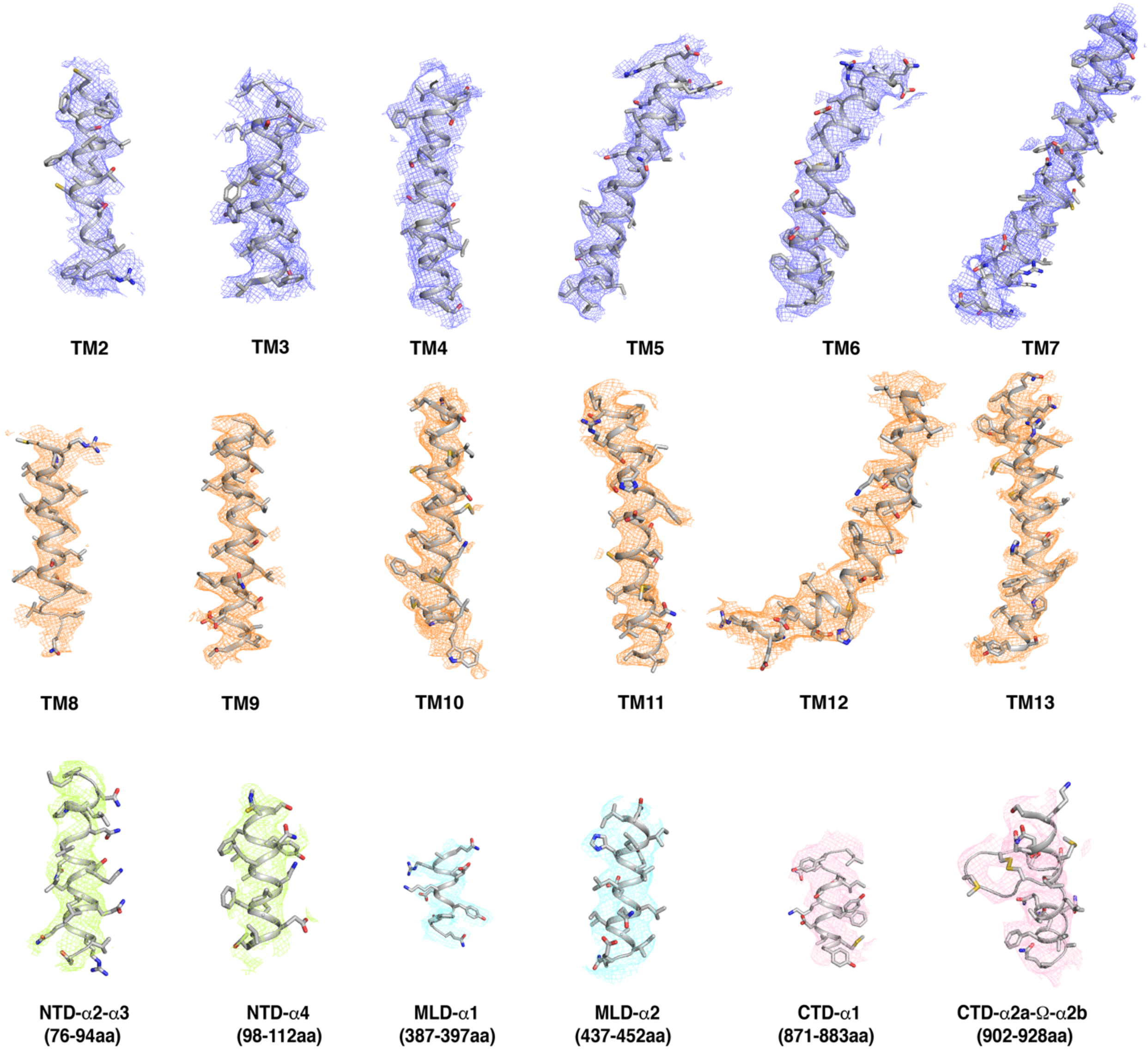

Supplementary Figure 7 Electron microscopy density of different portions of NPC1.

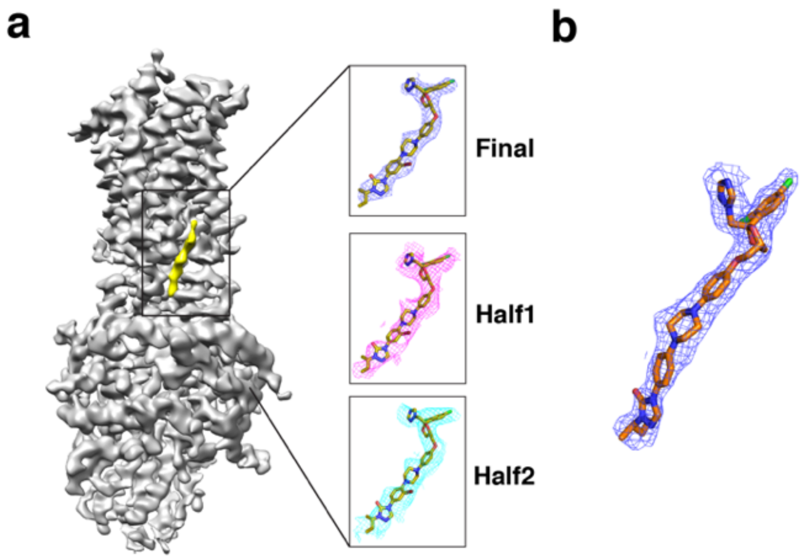

Supplementary Figure 8 The density of itraconazole in different maps.

a, The density of I-Br in the final map (blue), half1 map (pink) and half2 map (cyan). **b**, The density of itraconazole in crystal structure of lanosterol 14-α demethylase and itraconazole complex (PDB: 5EQB).

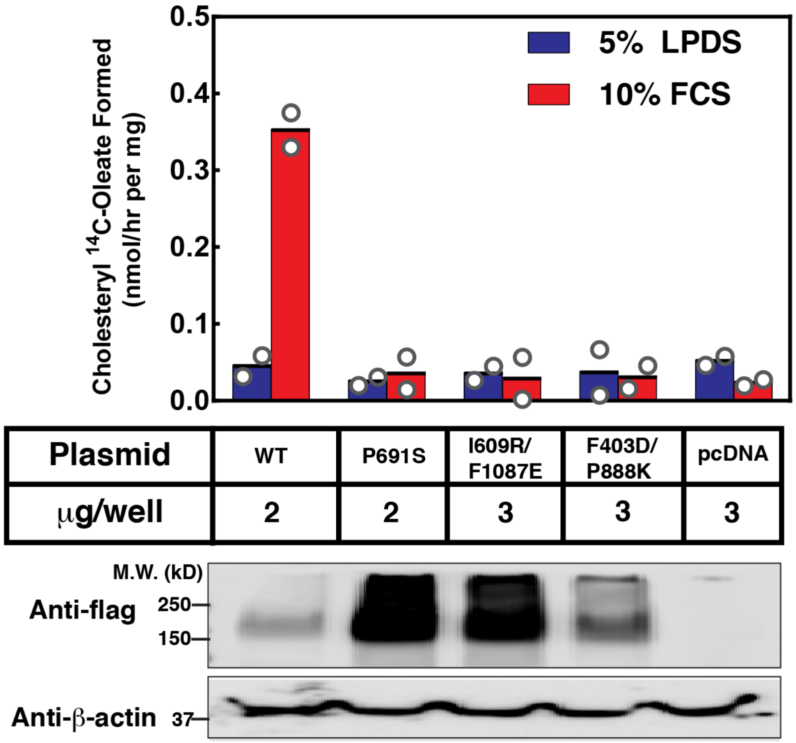

Supplementary Figure 9 Biological repeats of ACAT assays. Source data are provided as a Source Data file.

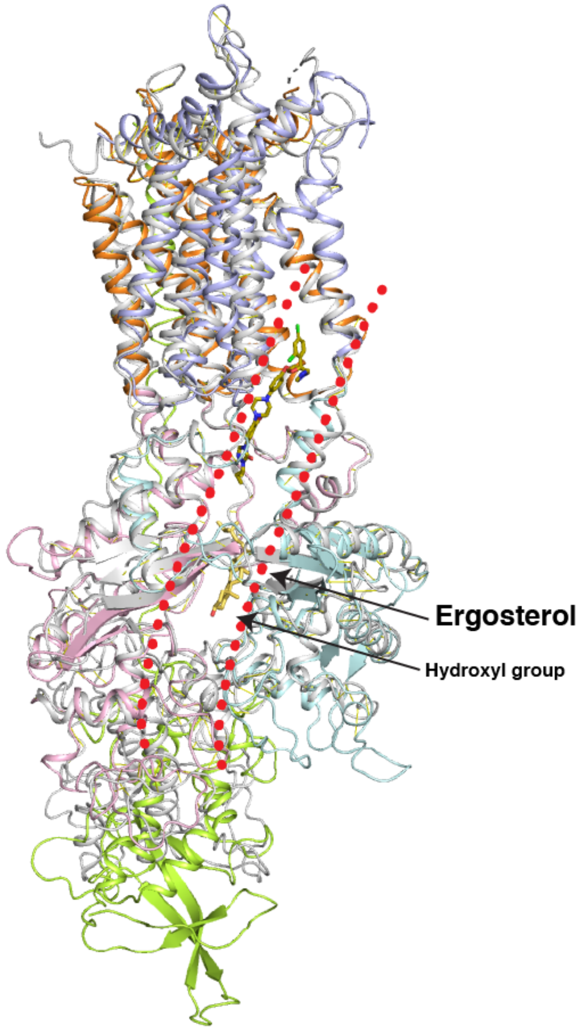

Supplementary Figure 10 Structural comparison of human NPC1 and yeast NPC1 (gray, PDB: 6R4L). An ergosterol presents in the putative tunnel with its hydrophobic side chain leading and its polar hydroxyl group following.

Supplementary Table 1 Statistics of data collection and refinement.

	I-Br–NPC1	Itraconazole-D–NPC1
**Data Collection**		
Microscope	Titan Krios (FEI)	Titan Krios (FEI)
Voltage (kV)	300	300
Detector	K3 Summit (Gatan)	K3 Summit (Gatan)
Pixel size (Å)	0.66	0.66
Defocus range (μm)	-1.2 to -2.4	-1.4 to -2.6
Movies	7564	9988
Frames/movie	30	30
Total dose (electrons/Å^2)	80	80
Number of particles	645,251	1,064,730
**Model composition**		
Non-hydrogen atoms	9138	
Protein residues	1161	
Ligand	1	
**Refinement**		
Number of particles for refinement	209,612	
Resolution (Å)	4.02	
RMS deviations		
Bond lengths (Å)	0.008	
Bond angles (°)	1.157	
Ramachandran plot (%)		
Favored	91.93	
Allowed	8.07	
Disallowed	0	
**Clashscore**	0.78	
**Molprobity score**	1.23	

Supplementary Table 2. Lists of primers used in this study.

**Primer name**	**Sequences (5' – 3')**
NPC1_W381E_FORWARD	ACCAATCCAGTTGACCTCGAGTCAGCCCCCAGCAGCCAG
NPC1_W381E_REVERSE	CTGGCTGCTGGGGGCTGACTCGAGGTCAACTGGATTGGT
NPC1_L613E_FORWARD	CGAAGTATTGAAGATGAAGAGAATCGTGAAAGTGACAG
NPC1_L613E_REVERSE	CTGTCACTTTCACGATTCTCTTCATCTTCAATACTTCG
NPC1_I685S_FORWARD	GTTGCCCTTGACCCTCAGCGTGATTGAAGTCATC
NPC1_I685S_REVERSE	GATGACTTCAATCACGCTGAGGGTCAAGGGCAAC
NPC1_Y1225E_FORWARD	CAAATTTTCCAGATATTCGAGTTCAGGATGTATTTG
NPC1_Y1225E_REVERSE	CAAATACATCCTGAACTCGAATATCTGGAAAATTTG
NPC1_I609R_FORWARD	TTCACTGCTGAACGAAGTCGTGAAGATGAACTAAATCGT
NPC1_I609R_REVERSE	ACGATTTAGTTCATCTTCACGACTTCGTTCAGCAGTGAA
NPC1_F1087E_FORWARD	TACAGTGTGTTTTATGTCGAATACGAACAGTACCTGACC
NPC1_F1087E_REVERSE	GGTCAGGTACTGTTCGTATTCGACATAAAACACACTGTA
NPC1_F403D_FORWARD	CAGCACTTTGGGCCTTTCGACCGGACGGAGCAGCTCATC
NPC1_F403D_REVERSE	GATGAGCTGCTCCGTCCGGTCGAAAGGCCCAAAGTGCTG
NPC1_P888K_FORWARD	TACCTGCATGCGGGTCCGAAAGTGTACTTTGTCCTGGAG
NPC1_P888K_REVERSE	CTCCAGGACAAAGTACACTTTCGGACCCGCATGCAGGTA

Supplementary method

Synthesis of Br-labeled itraconazole (I-Br)

Anhydrous solvents were obtained by passing them through commercially available alumina columns (Innovative technology, Inc., MA). All solvents were of HPLC or ACS grade. All reagents were commercial compounds of the highest purity available. Analytical thin layer chromatography (TLC) was performed on aluminium plates with Merck Kieselgel 60F254 and visualized by UV irradiation (254 nm) or by staining with a solution of potassium permanganate. Flash column chromatography was carried out using Merck Kieselgel 60 (230– 400 mesh) under pressure. ^{1}H NMR spectra were recorded in $CDCl_3$ and DMSO-d_6 at ambient temperature on a Varian Inova-400 spectrometer at 400 MHz with residual protic solvent as the internal reference ($CDCl_3$, d_H = 7.26 ppm; DMSO-d_6, d_H = 2.50 ppm); chemical shifts (*d*) are given in parts per million (ppm), and coupling constants (*J*) are given in Hertz (Hz). The proton spectra are reported as follows: d (multiplicity, coupling constant *J*, number of protons). The following abbreviations were used to explain the multiplicities: app = apparent, b = broad, d = doublet, dd = doublet of doublets, ddd = doublet of doublet of doublets, dddd = doublet of doublet of doublet of doublets, m = multiplet, s = singlet, t = triplet. ^{13}C NMR spectra were recorded in $CDCl_3$, DMSO-d_6 at ambient temperature on the same spectrometer at 100 MHz with the central peak of $CDCl_3$ (d_C = 77.0 ppm), DMSO-d6 (d_C = 39.4 ppm) as the internal reference. Electrospray ionization mass spectra (ESI-MS) were recorded on a Shimadzu 2010-LCMS. HRMS were performed on a Shimadzu IT-TOF.

Scheme 1. Synthesis of compound 6

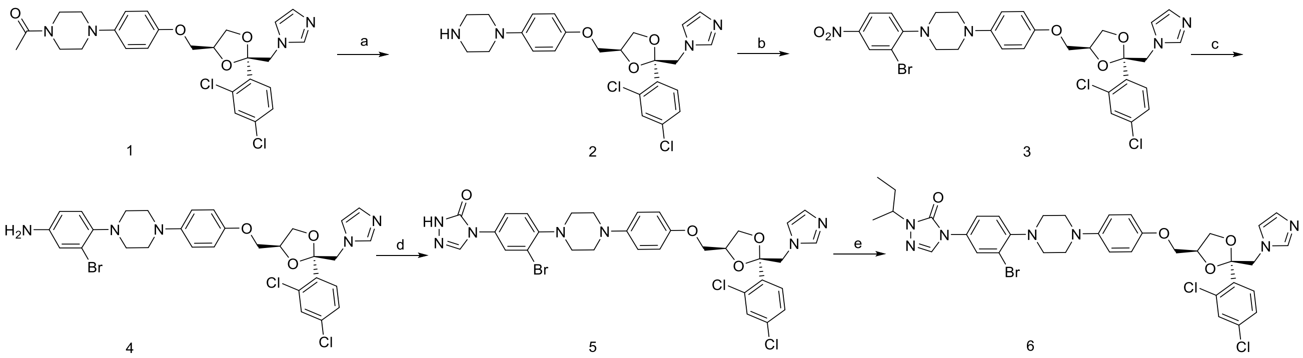

Reagents and conditions: a) KOH, H_2O, MeOH, reflux; b) 2-bromo-1-chloro-4-nitrobenzene, K_2CO_3, DMF, 110 ^oC; c) Fe, NH_4Cl, MeOH, H_2O, reflux; d) methyl hydrazinocarboxylate, trimethyl orthoformate, TsOH, NaOMe, MeOH; e) 2-bromobutane, K_2CO_3, DMF, 110 ^oC.

The synthesis of bromo-itraconazole (**6**) as depicted in Scheme 1 is based on a modified route described in [2].

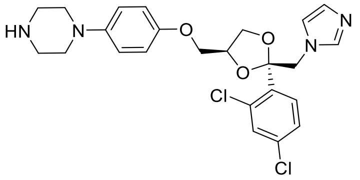

(±)-*cis*-2-((1H-imidazol-1-yl)methyl)-2-(2,4-dichlorophenyl)-1,3-dioxolan-4-yl)methoxy)phenyl) piperazine (2): To a solution of Ketoconazole **1** (531 mg, 1.0 mmol) in 4 mL of MeOH was added KOH (2.32 g in 2 mL of H_2O) at room temperature. The solution was heated to reflux and stirred for 6 hr under reflux, cooled to room temperature followed by dilution with 10 mL of water. The mixture was filtered. The solid obtained was washed with 15 mL of water, dried to provide known compound **2** (390 mg, 0.80 mmol, 80%). The spectroscopic data was in agreement with published data [2].

^{1}H NMR (400 MHz, $CDCl_3$) δ 7.55 (d, *J* = 8.4 Hz, 1H), 7.48 (s, 1H), 7.44 (d, *J* = 2.0 Hz, 1H), 7.23 (dd, *J* = 8.4, 2.0 Hz, 1H), 6.95 (d, *J* = 11.2 Hz, 2H), 6.86 (d, *J* = 9.2 Hz, 2H), 6.74 (d, *J* = 9.2 Hz, 2H), 4.48 (d, *J* = 14.4 Hz, 1H), 4.38 (d, *J* = 14.4 Hz, 1H), 4.32 (tt, *J* = 6.4, 5.6 Hz, 1H), 3.85 (dd, *J* = 8.4, 6.4 Hz, 1H), 3.71 (dd, *J* = 8.8, 4.8 Hz, 2H), 3.28 (dd, *J* = 9.6, 6.8 Hz, 1H), 3.05-2.95 (m, 8H), 1.97 (s, 1H); **^{13}C NMR** (100 MHz, $CDCl_3$) δ 152.2, 146.6, 138.7, 135.8, 134.5, 132.9, 131.3, 129.5, 128.5, 127.2, 121.1, 118.0, 115.1, 107.9, 74.7, 67.6, 67.5, 51.72, 51.2, 46.2; **MS (ESI):** calcd. for $C_{24}H_{27}Cl_2N_4O_3Na$ $[M+H]^+$ 489.1, found 489.2.

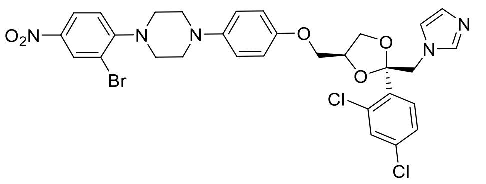

(±)-*cis*-2-((1H-imidazol-1-yl)methyl)-2-(2,4-dichlorophenyl)-1,3-dioxolan-4-yl)methoxy)phenyl)-4-(2-bromo-4-nitrophenyl)piperazine (3): To a solution of compound **2** (110 mg, 0.22 mmol) in 3 mL of DMF was added 2-bromo-1-chloro-4-nitrobenzene (106 mg, 0.44 mmol) and K_2CO_3 (45 mg, 0.33 mmol) at room temperature. The solution was heated to 110 ^{o}C and stirred for overnight at 110 ^{o}C, cooled to room temperature followed by dilution with EtOAc (30 mL) and washed with brine (3 × 3 ml). The organic layer was dried (Na_2SO_4) and concentrated. The residue was purified by flash chromatography (silica gel; CH_2Cl_2:MeOH = 15:1) to provide compound **3** (120 mg, 0.17 mmol, 79%).

^{1}H NMR (400 MHz, $CDCl_3$) δ 8.46 (d, *J* = 2.4 Hz, 1H), 8.16 (dd, *J* = 8.8, 2.4 Hz, 1H), 7.58 (d, *J* = 8.4 Hz, 1H), 7.57 (s, 1H), 7.46 (d, *J* = 2.0 Hz, 1H), 7.29 -7.21 (m, 1H), 7.10 (d, *J* = 8.8 Hz, 1H), 6.99 (d, *J* = 10.4 Hz, 2H), 6.93 (d, *J* = 8.8 Hz, 2H), 6.78 (d, *J* = 8.8 Hz, 2H), 4.51 (d, *J* = 14.8 Hz, 1H), 4.41 (d, *J* = 14.8 Hz, 1H), 4.35 (dt, *J* = 11.2, 6.0 Hz, 1H), 3.87 (dd, *J* = 7.2, 7.2 Hz,

1H), 3.72 (dt, J = 9.6, 5.2 Hz, 2H), 3.35 (t, J = 4.8Hz, 4H), 3.33-3.30 (m, 1H), 3.27 (t, J = 4.8Hz, 4H); **^{13}C NMR** (100 MHz, $CDCl_3$) δ 156.1, 152.6, 145.7, 142.6, 138.6, 135.9, 134.4, 132.9, 131.3, 129.8, 129.5, 127.9, 127.2, 124.0, 121.2, 119.8, 118.4, 117.7, 115.2, 107.9, 74.8, 67.6, 67.5, 51.34 51.1, 50.5; **HRMS (ESI):** calcd. for $C_{30}H_{29}Cl_2BrN_5O_5$ $[M+H]^+$ 688.0724, found 688.0732.

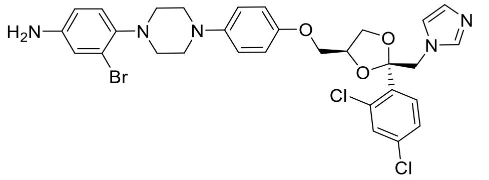

(±)-*cis*-2-((1H-imidazol-1-yl)methyl)-2-(2,4-dichlorophenyl)-1,3-dioxolan-4yl)methoxy) phenyl)piperazin -1-yl)-3-bromoaniline (4): To a solution of compound **3** (100 mg , 0.14 mmol) and iron (39 mg, 0.7 mmol) in MeOH (3 mL) was added saturated NH_4Cl solution 1 mL. The solution was stirred for overnight under reflux, cooled to room temperature followed by dilution with EtOAc (30 mL) and washed with brine (3 × 3 ml). The organic layer was dried (Na_2SO_4) and concentrated. The residue was purified by flash chromatography (silica gel; CH_2Cl_2:MeOH = 15:1) to provide compound **4** (90 mg, 0.12 mmol, 94%).

^{1}H NMR (400 MHz, $CDCl_3$) δ 7.57 (d, J = 8.4 Hz, 1H), 7.53 (s, 1H), 7.46 (d, J = 2.0 Hz, 1H), 7.25 (dd, J = 8.4, 2.0 Hz, 1H), 7.04-6.89 (m, 6H), 6.77 (d, J = 8.8 Hz, 2H), 6.62 (dd, J = 8.4, 2.8 Hz, 1H), 4.51 (d, J = 14.8 Hz, 1H), 4.40 (d, J = 14.8 Hz, 1H), 4.38-4.29 (m, 1H), 3.87 (dd, J = 8.4, 6.8 Hz, 1H), 3.80-3.69 (m, 2H), 3.38-3.27 (m, 3H), 3.23 (t, J = 4.8 Hz, 4H), 3.13 (t, J = 4.8 Hz, 4H); **^{13}C NMR** (100 MHz, $CDCl_3$) δ 152.2, 146.2, 143.4, 142.0, 138.7, 135.8, 134.5, 132.9, 131.3, 129.5, 128.3, 127.2, 121.6, 121.1, 121.0, 120.0, 118.1, 115.1, 114.8, 107.9, 74.8, 67.7, 67.5, 52.3, 51.3, 50.9; **MS (ESI):** calcd. for $C_{30}H_{31}BrCl_2N_5O_3$ $[M+H]^+$ 658.1, found 658.1.

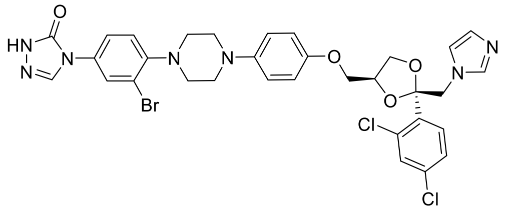

(±)-*cis*-2-((1H-imidazol-1-yl)methyl)-2-(2,4-dichlorophenyl)-1,3-dioxolan-4-yl)methoxy)phenyl) piperazin-1-yl)-3-bromophenyl)-2,4-dihydro-3H-1,2,4-triazol-3-one (5): To a solution of compound **4** (36 mg, 0.05 mmol), methyl hydrazinocarboxylate (10 mg, 0.12 mmol) and TsOH (2 mg, 0.01 mmol) in MeOH (3 mL) was added trimethyl orthoformate (11 mg, 0.11 mmol). The solution was stirred at 60°C for overnight. After cooling to room temperature, NaOMe (15 mg, 0.3 mmol) was added and the mixture was stirred at room temperature for another 4 hr. The reaction was quenched by adding aq. NH_4Cl (4 mL), extracted with CH_2Cl_2 (3 x 15 mL). The combined organic layers were washed with brine (3 × 3 ml), dried (Na_2SO_4), and concentrated. The residue was purified by column chromatography (silica gel; MeOH:CH_2Cl_2 = 1:10) to yield compound **5** (28 mg, 0.04 mmol, 77%).

^{1}H NMR (400 MHz, DMSO-d_6) δ 12.00 (s, 1H), 8.36 (s, 1H), 7.98 (d, J = 2.4 Hz, 1H), 7.72-7.63 (m, 2H), 7.56 (d, J = 8.4 Hz, 1H), 7.47 (s, 1H), 7.44 (dd, J = 8.4, 2.0 Hz, 1H), 7.32 (d, J = 8.8 Hz, 1H), 7.00 (s, 1H), 6.94 (d, J = 8.8 Hz, 2H), 6.80 (s, 1H), 6.79(d, J = 8.8 Hz, 2H), 4.52 (dd, J = 18.4, 14.8 Hz, 2H), 4.33 (dt, J = 11.2, 5.6 Hz, 1H), 3.85 (dd, J = 8.4, 6.8 Hz, 1H), 3.69-3.59 (m, 2H), 3.52 (dd, J = 10.4, 5.2 Hz, 1H), 3.19 (t, J = 4.0 Hz, 4H), 3.13 (t, J = 4.0 Hz, 4H); **^{13}C NMR** (100 MHz, DMSO-d_6) δ 153.3, 152.2, 150.6, 149.2, 146.0, 138.9, 136.6, 135.6, 134.9, 132.8, 131.0, 130.5, 128.0, 127.7, 126.8, 122.2, 122.0, 121.5, 119.3, 117.8, 115.5, 108.1, 75.0, 68.1, 67.1, 51.8, 50.9, 50.1; **HRMS (ESI):** calcd. for $C_{32}H_{31}Cl_2BrN_7O_4$ $[M+H]^+$ 726.0992, found 726.1011.

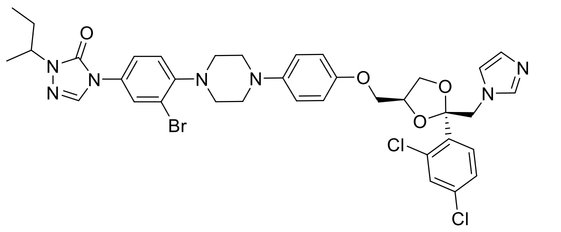

(±)-*cis*-2-((1H-imidazol-1-yl)methyl)-2-(2,4-dichlorophenyl)-1,3-dioxolan-4-yl)methoxy)phenyl) piperazin-1-yl)-3-bromophenyl)-2-(sec-butyl)-2,4-dihydro-3H-1,2,4-triazol-3-one (6): To a solution of compound **5** (5 mg, 0.007 mmol) in DMF (1 mL) was added 2-bromobutane (14 mg, 0.1 mmol) and K_2CO_3 (14 mg, 0.1 mmol) at room temperature. The solution was heated to 110 ^oC and stirred for overnight at 110 ^oC, cooled to room temperature followed by dilution with EtOAc (20 mL) and washed with brine (3 × 3 ml). The organic layer was dried (Na_2SO_4) and concentrated. The residue was purified by flash chromatography (silica gel; CH_2Cl_2:EtOAc = 3:1) to provide product **6** (4 mg, 0.005 mmol, 72%).

^{1}H NMR (400 MHz, $CDCl_3$) δ 7.79 (d, *J* = 2.4 Hz, 1H), 7.64 (s, 1H), 7.57 (d, *J* = 8.4 Hz, 1H), 7.54 (s, 1H), 7.51 (dd, *J* = 8.4, 2.4 Hz, 1H), 7.46 (d, *J* = 2.0 Hz, 1H), 7.25 (dd, *J* = 8.4, 2.0 Hz, 1H), 7.16 (d, *J* = 8.8 Hz, 1H), 6.99 (d, *J* = 12.4 Hz, 2H), 6.94 (d, *J* = 9.2 Hz, 2H), 6.78 (d, *J* = 9.2 Hz, 2H), 4.51 (d, *J* = 14.8 Hz, 1H), 4.39 (d, *J* = 14.8 Hz, 1H), 4.38-4.31 (m, 1H), 4.31-4.23 (m, 1H), 3.87 (dd, *J* = 8.4, 6.4 Hz, 1H), 3.73 (dd, *J* = 8.8, 4.8 Hz, 2H), 3.32 (dd, *J* = 9.6, 6.8 Hz, 1H), 3.31-3.24 (m, 4H), 3.24-3.15 (m, 4H), 1.93-1.79 (m, 1H), 1.78-1.65 (m, 1H), 1.38 (d, *J* = 6.8 Hz, 3H), 0.89 (t, *J* = 7.2 Hz, 3H); **^{13}C NMR** (100 MHz, $CDCl_3$) δ 152.4, 151.5, 149.8, 146.0, 138.7, 135.8, 134.5, 133.2, 132.9, 131.3, 129.7, 129.5, 128.2, 127.5, 127.2, 122.0, 121.2, 120.1, 118.3, 115.2, 109.9, 107.9, 74.8, 67.6, 67.5, 52.8, 51.7, 51.3, 50.7, 28.3, 19.2, 10.7; **HRMS (ESI):** calcd. for $C_{36}H_{38}Cl_2BrN_7O_4$ $[M+Na]^+$ 804.1438, found 804.1441.

Reference:

1 Shi, W., Nacev, B. A., Bhat, S. & Liu, J. O. Impact of Absolute Stereochemistry on the Antiangiogenic and Antifungal Activities of Itraconazole. *ACS medicinal chemistry letters* **1**, 155-159, doi:10.1021/ml1000068 (2010).

2 Pace, J. R. *et al.* Repurposing the Clinically Efficacious Antifungal Agent Itraconazole as an Anticancer Chemotherapeutic. *Journal of medicinal chemistry* **59**, 3635-3649, doi:10.1021/acs.jmedchem.5b01718 (2016).
