# Supplementary material for: Structural basis for itraconazole-mediated NPC1 inhibition
Source: Nat Commun. 2020 Jan 9;11:152. doi: 10.1038/s41467-019-13917-5 (PMC6952396; doi:10.1038/s41467-019-13917-5)
Supplement: Supplementary file 2 — Reporting Summary [file 41467_2019_13917_MOESM2_ESM.pdf]

nature research

Corresponding author(s): Xiaochun Li

Last updated by author(s): Nov 30, 2019

Reporting Summary

Nature Research wishes to improve the reproducibility of the work that we publish. This form provides structure for consistency and transparency in reporting. For further information on Nature Research policies, see Authors & Referees and the Editorial Policy Checklist .

Statistics

For all statistical analyses, confirm that the following items are present in the figure legend, table legend, main text, or Methods section.

n/a	Confirmed	
x		The exact sample size (*n*) for each experimental group/condition, given as a discrete number and unit of measurement
	x	A statement on whether measurements were taken from distinct samples or whether the same sample was measured repeatedly
x		The statistical test(s) used AND whether they are one- or two-sided<br>*Only common tests should be described solely by name; describe more complex techniques in the Methods section.*
x		A description of all covariates tested
x		A description of any assumptions or corrections, such as tests of normality and adjustment for multiple comparisons
x		A full description of the statistical parameters including central tendency (e.g. means) or other basic estimates (e.g. regression coefficient) AND variation (e.g. standard deviation) or associated estimates of uncertainty (e.g. confidence intervals)
x		For null hypothesis testing, the test statistic (e.g. *F*, *t*, *r*) with confidence intervals, effect sizes, degrees of freedom and *P* value noted<br>*Give P values as exact values whenever suitable.*
x		For Bayesian analysis, information on the choice of priors and Markov chain Monte Carlo settings
x		For hierarchical and complex designs, identification of the appropriate level for tests and full reporting of outcomes
x		Estimates of effect sizes (e.g. Cohen's *d*, Pearson's *r*), indicating how they were calculated

Our web collection on statistics for biologists contains articles on many of the points above.

Software and code

Policy information about availability of computer code

Data collection	SerialEM
Data analysis	Graphpad, Phenix, COOT, PyMOL, MolProbity, Relion-3

For manuscripts utilizing custom algorithms or software that are central to the research but not yet described in published literature, software must be made available to editors/reviewers. We strongly encourage code deposition in a community repository (e.g. GitHub). See the Nature Research guidelines for submitting code & software for further information.

Data

Policy information about availability of data

All manuscripts must include a data availability statement. This statement should provide the following information, where applicable:

- Accession codes, unique identifiers, or web links for publicly available datasets
- A list of figures that have associated raw data
- A description of any restrictions on data availability

The 3D cryo-EM density maps have been deposited in the Electron Microscopy Data Bank under the accession numbers EMD-20834. Atomic coordinates for the atomic model have been deposited in the Protein Data Bank under the accession numbers 6UOX. All other data is available from the corresponding authors upon reasonable request.

Field-specific reporting

Please select the one below that is the best fit for your research. If you are not sure, read the appropriate sections before making your selection.

Life sciences study design

All studies must disclose on these points even when the disclosure is negative.

Sample size	No statistical method was used to determine sample size.
Data exclusions	No data was excluded.
Replication	Each experiment was reproduced at least three times on separate occasions. Experimental findings were reliably reproduced.
Randomization	The study did not involve animals or human research participants thus samples were not randomized for the experiments.
Blinding	The study did not involve animals or human research participants thus thus no blinding was used.

Reporting for specific materials, systems and methods

We require information from authors about some types of materials, experimental systems and methods used in many studies. Here, indicate whether each material, system or method listed is relevant to your study. If you are not sure if a list item applies to your research, read the appropriate section before selecting a response.

Materials & experimental systems

n/a	Involved in the study	
☐	☒	Antibodies
☐	☒	Eukaryotic cell lines
☒	☐	Palaeontology
☒	☐	Animals and other organisms
☒	☐	Human research participants
☒	☐	Clinical data

Methods

n/a	Involved in the study	
☒	☐	ChIP-seq
☒	☐	Flow cytometry
☒	☐	MRI-based neuroimaging

Antibodies

Antibodies used	Rabbit monocolonal IgG against human NPC1 (ab1341113, abcam); Mouse monoclonal IgG against Flag epitope DDDDK (Cat. No. M185, MBL International Corp.); Rabbit monocolonal IgG against Flag (F7425, Sigma-Aldrich); Mouse monoclonal IgG aginst LAMP2 (555803, BD Biosciences); Goat anti-rabbit IgG conjugated to AlexaFluor 488 (A-11008, Invitrogen); Goat anti-mouse IgG conjugated to AlexaFluor 594 (A-11005, Invitrogen); Mouse monoclonal HRP-conjugated IgG against b-actin (12262, Cell Signaling Technology); Horse anti-mouse IgG conjugated to HRP (7076, Cell Signaling Technology); Goat anti-rabbit IgG conjugated to HRP (7074, Cell Signaling Technology)
Validation	Rabbit monocolonal IgG against human NPC1 recognizes Mouse, Rat and Human NPC1, applying Flow Cyt, WB, IHC-P and ICC/IF; Mouse monoclonal IgG against Flag epitope DDDDK reacts with N-terminal, Internal and C-terminal DDDDK-tagged(DYKDDDDK) proteins, applying FCM, ICC, IP and WB. Rabbit monocolonal IgG against Flag recognizes the FLAG epitope located on FLAG-tagged fusion proteins at the N-terminus or C-terminus, applying dot blot, immunoblotting, immunoprecipitation and immunocytochemistry assays. Mouse monoclonal IgG aginst LAMP2 recognizes Human LAMP2, applying Intracellular staining (flow cytometry). Mouse monoclonal HRP-conjugated IgG against b-actin recognizes Human, Mouse, Rat, Hamster, Monkey and Dog b-actin, applying WB.

Eukaryotic cell lines

Policy information about cell lines

Cell line source(s)	The protein was expressed from HEK-293S GnTI– (ATCC). CHO (ATCC) and NPC1-KO cells (from Laura Liscum, Tufts University) were used for the ACAT assay.
Authentication	No further authentication was performed for commercially available cell lines.
Mycoplasma contamination	periodically test negative
Commonly misidentified lines (See ICLAC register)	None of the cell lines used is listed in the database of commonly misidentified cell lines maintained by ICLAC.
